# Bioengineered corneal tissue for minimally invasive vision restoration in advanced keratoconus in two clinical cohorts

**DOI:** 10.1038/s41587-022-01408-w

**Published:** 2022-08-11

**Authors:** Mehrdad Rafat, Mahmoud Jabbarvand, Namrata Sharma, Maria Xeroudaki, Shideh Tabe, Raha Omrani, Muthukumar Thangavelu, Anthony Mukwaya, Per Fagerholm, Anton Lennikov, Farshad Askarizadeh, Neil Lagali

**Affiliations:** 1 LinkoCare Life Sciences AB, Linköping, Sweden; 2 Department of Biomedical Engineering, Linköping University, Linköping, Sweden; 3 Farabi Eye Hospital, Tehran University of Medical Sciences, Tehran, Iran; 4 R.P. Centre for Ophthalmic Sciences, All India Institute of Medical Sciences, New Delhi, India; 5 Division of Ophthalmology, Department of Biomedical and Clinical Sciences, Linköping University, Linköping, Sweden; 6 Department of Optometry, Faculty of Rehabilitation Sciences, Tabriz University of Medical Sciences, Tabriz, Iran

**Keywords:** Translational research, Biomedical materials, Tissue engineering, Implants

## Abstract

Visual impairment from corneal stromal disease affects millions worldwide. We describe a cell-free engineered corneal tissue, bioengineered porcine construct, double crosslinked (BPCDX) and a minimally invasive surgical method for its implantation. In a pilot feasibility study in India and Iran (clinicaltrials.gov no. NCT04653922), we implanted BPCDX in 20 advanced keratoconus subjects to reshape the native corneal stroma without removing existing tissue or using sutures. During 24 months of follow-up, no adverse event was observed. We document improvements in corneal thickness (mean increase of 209 ± 18 µm in India, 285 ± 99 µm in Iran), maximum keratometry (mean decrease of 13.9 ± 7.9 D in India and 11.2 ± 8.9 D in Iran) and visual acuity (to a mean contact-lens-corrected acuity of 20/26 in India and spectacle-corrected acuity of 20/58 in Iran). Fourteen of 14 initially blind subjects had a final mean best-corrected vision (spectacle or contact lens) of 20/36 and restored tolerance to contact lens wear. This work demonstrates restoration of vision using an approach that is potentially equally effective, safer, simpler and more broadly available than donor cornea transplantation.

## Abstract (web summary)

A bioengineered cornea made of collagen restores vision in a pilot clinical study.

## Main

Loss of corneal transparency and poor refractive function are among the leading causes of blindness globally^1–4^. Although corneal blindness can be treatable by transplantation, an estimated 12.7 million people await a donor cornea, with one cornea available for every 70 needed^3^. With an incidence of over 1 million new cases of corneal blindness annually^5^, the severe shortage of donor corneas presents an unequal burden of blindness heavily skewed towards low- and middle-income countries (LMICs) in Asia, Africa and the Middle East^2,3^. Over half of the world’s population does not have access to corneal transplantation owing to a lack of infrastructure for tissue donation, harvesting, testing and eye banking in LMICs^1,3^. The access problem is complex, involving economic, cultural, technological, political and ethical barriers^2,4^. Additionally, infectious diseases and pandemics bring donor tissue procurement and use to a virtual standstill, necessitating further measures to ensure donor tissue safety^6,7^.

For these reasons, intense research effort has focused on bioengineering tissue for corneal transplantation^8–10^. To date, however, no biotechnological advance has been able to address the burden of corneal blindness or improve access to transplantable corneal tissue. In many parts of the world including Europe and Australia, keratoconus—a corneal disease characterized by stromal thinning, weakening and scarring^11^—is the leading indication for corneal transplantation^2,12^. Keratoconus affects both men and women and all ethnic groups, with highest prevalence reported in China (0.9%, or 12.5 million)^13^, India (2.3%, or 30 million)^14^ and Iran (4% of the rural population, or 3.4 million)^15^.

Keratoconus is progressive, but with a complex etiology that is not well understood. With proper screening and access to specialist care, keratoconus progression can be detected and halted in its early stages while vision is still good; however, if not addressed early and in LMICs where keratoconus is highly prevalent and access to healthcare is limited, the disease often progresses. In advanced stages, it requires transplantation to prevent blindness, using techniques such as penetrating keratoplasty (PK) or deep anterior lamellar keratoplasty (DALK)^16–19^. These techniques, however, are subject to the limited supply of donor corneas, risk of graft rejection, post-operative complications associated with sutures and wound healing, risk of corneal neovascularization and/or infection, high astigmatism after suture removal, need for long-term immunosuppression and necessity for long-term patient follow-up^20^. To partially address these issues, newer and less invasive techniques such as stromal lenticule addition keratoplasty^21^ and Bowman layer transplantation^22^ have been introduced. While promising and still developing, these techniques stabilize the condition but offer only marginal vision improvement^21,23^, and rely on availability of donor corneas and tissue banking infrastructure and are thus inapplicable in many regions of the world.

To address these limitations, we bioengineered a cell-free implantable medical device as a substitute for human corneal stromal tissue. As a raw material we used natural type I collagen, the main protein in the human cornea^24^. For an abundant yet sustainable and cost-effective supply of collagen, we used medical-grade collagen sourced from porcine skin, a purified byproduct from the food industry already used in FDA-approved medical devices for glaucoma surgery^25^ and as a wound dressing^26^. In a previous clinical study^27,28^, we evaluated implants engineered from recombinant human collagen that had several limitations: the collagen could be produced only in small quantities, implants were mechanically weak and required invasive suturing, implants were not evaluated for long-term stability, and surgery was invasive and led to a strong wound-healing response and partial implant melting. Here we addressed these limitations by using type I medical-grade porcine dermal collagen, developing a new method of double crosslinking to improve implant strength and stability, and using a new minimally invasive surgical implantation technique to promote corneal thickening, reshaping and rapid wound healing.

Pure collagen is a soft material prone to degradation, so we applied dual chemical and photochemical crosslinking to form a transparent implantable hydrogel, termed the bioengineered porcine construct, double crosslinked (BPCDX). BPCDX is an improvement on our earlier porcine collagen-based materials^29–31^ that has additionally been photochemically crosslinked with the UVA–riboflavin crosslinking procedure^32^. BPCDX, fabricated in a good manufacturing practices (GMP)-certified clean room according to stringent quality processes, was tested to evaluate optical and mechanical properties, enzymatic degradation and cell compatibility and underwent a panel of third-party certified medical device tests compliant with ISO standards to assess biocompatibility, toxicity, carcinogenicity, sensitization and irritation using in vitro and in vivo assays in mice, guinea pigs and rabbits.

A further challenge is to provide devices to different regions and potentially to rural areas without biobanking or storage and tissue preparation facilities. We addressed this by developing compatible packaging and sterilization processes and testing packaged devices in accelerated and real-time ISO shelf-life stability studies, to compare optical, mechanical, chemical and sterility properties of the fully packaged BPCDX as-made and after storage for up to two years.

Conventional transplantation techniques for advanced keratoconus remove and/or damage corneal epithelium, endothelium and nerves. On the basis of earlier studies in rabbits^29,31^, we developed a minimally invasive surgery for advanced keratoconus, inserting a thick, large-diameter BPCDX into an intrastromal pocket within the recipient cornea to counteract pathologic stromal thinning and normalize refraction by reshaping the central and peripheral cornea, without removing recipient tissue. The intrastromal surgery is suture-free and leaves corneal nerves and cellular layers intact, promoting rapid wound healing^29^. We specifically adapted previous intrastromal methods^21,33,34^ to use a single corneal incision half the size of previous techniques^21,34^ without disrupting the sclera or anterior chamber^22^, to significantly thicken and reshape the central cornea by inserting a 280–440-µm-thick BPCDX to achieve substantial flattening (>10 diopters (D)) of the steepest corneal curvature in keratoconus.

We first evaluated BPCDX implantation by this intrastromal method in a minipig model of advanced keratoconus, using surgical tools and protocols that different surgeons could replicate. To obtain human safety and feasibility data to justify a controlled clinical trial, we undertook a pilot feasibility study in India and Iran. Here we report safety and efficacy results in the first 20 advanced keratoconus subjects receiving the BPCDX. No intra- or post-operative complications or adverse events were noted in any subject during 24 months of clinical follow-up. Significant and stable corneal thickening and flattening of keratometry, maintenance of corneal transparency and improvement of best-corrected visual acuity (BCVA) by a mean of 7.6 logMAR lines to a mean of 20/58 in Iran and by a mean of 15.1 logMAR lines to a mean of 20/26 in India was achieved. These represent equivalent outcomes to standard corneal transplantation but with a simpler surgical technique and without the need for human donor tissue or tissue banking infrastructure.

## Results

### Manufacturing of collagen scaffolds

**Fig. 1:**
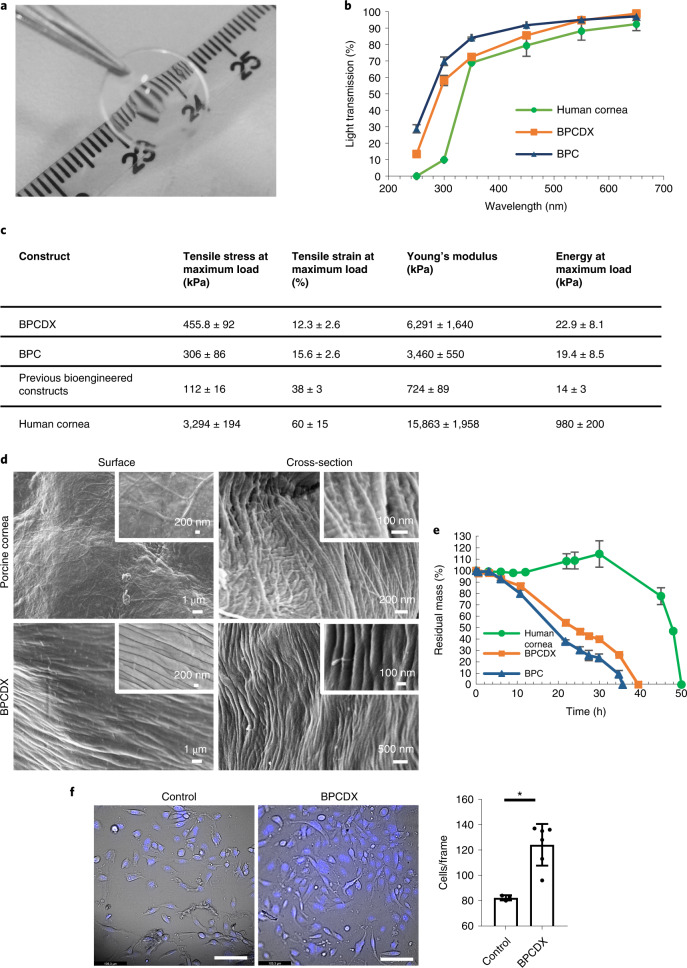
Biomaterial properties of BPCDX. **a**, Appearance of BPCDX, indicating transparency and refractive nature of the curved device. **b**, Light transmission through 550-µm-thick samples of BPCDX, single-crosslinked BPC and the human cornea. The human cornea contains a layer of epithelial cells which absorb UV light^61^, whereas the bioengineered materials are cell-free. Data shown represent mean and standard deviation of measurements from three independent samples. **c**, Mechanical properties of BPCDX relative to single-crosslinked BPC and previously published data of bioengineered constructs made from porcine collagen^29,30^, with human cornea reference values^30^ included for comparison. Data values for BPCDX represent mean and standard deviation of measurements from 22 independent samples per test (taken across different production batches, 550-µm-thick ‘dog-bone’ specimens). **d**, Scanning electron microscope images of the surface and bulk (cross-section) structure of BPCDX and a porcine cornea, indicating tightly packed collagen fibrils in BPCDX with diameter slightly thicker than the native porcine cornea (representative images from three samples per cornea type with similar results). **e**, Degradation of BPCDX, single-crosslinked BPC and a human donor cornea in 1 mg ml^−1^ collagenase (data represent mean and standard deviation of measurements from three independent samples for bioengineered materials (550 µm thick, 12 mm diameter) and two independent samples of human donor cornea). **f**, HCE-2 human corneal epithelial cell attachment and growth on BPCDX relative to the control culture plate surface after 16 days of culture. Cells adhered to BPCDX, with NucBlue staining indicating nuclei and morphology of live, viable cells in brightfield mode. BPCDX had greater cell density than cell culture plasticware (three control samples, six BPCDX samples; error bars represent mean and standard deviation, *P* = 0.003, two-sided independent *t*-test). Scale bars, 100 µm. Source Data

BPCDX is a corneal implant manufactured from purified medical-grade type I porcine collagen produced under GMP-compliant processes and conditions. No cells or viable biological material are present within BPCDX, and it is a Class III medical device designed to mimic properties of the natural cornea. The collagen in BPCDX is double crosslinked, both chemically and photochemically, imparting strength and resistance to degradation. The crosslinkers do not become integrated within the final device as they are water-soluble and rinsed out of the implant during manufacturing, resulting in an entirely natural, transparent hydrogel (Fig. 1a).

### Optical and mechanical properties of scaffolds

In comparison to single-crosslinked BPC^29,31^ and an earlier reported bioengineered porcine collagen-based cornea^30^, BPCDX transmits visible light similarly to the human cornea^35^ and exhibits improved mechanical properties without sacrificing transparency (Fig. 1b,c). Notably, the BPCDX stiffness (Young’s modulus) is significantly greater than an earlier collagen-based biomaterial we evaluated in humans^28^ (*P* < 0.0001, Supplementary Table 1) and is within the reported range of the healthy human cornea^28,36,37^.

### Microstructure by scanning electron microscopy

Surface and cross-sectional profiles of BPCDX and the porcine cornea obtained by scanning electron microscopy (SEM; Fig. 1d) revealed packed collagen fibrils with 300–1,000 nm diameter on BPCDX surface, while fibrils within the bulk had about 100 nm diameter. These results confirm previous observations of surface layers with differing morphology than the bulk^29^, with collagen fibril diameter corresponding to the literature^38^. Fibrils on the porcine cornea surface were harder to identify owing to a basement membrane Bowman complex of randomly-oriented fibrils; however, these few fibrils had 200–500 nm diameter, while in the bulk 50–100 nm diameter was observed. Overall, BPCDX consisted of aligned fibrillar collagen with slightly larger fibril size and similar tight packing as the porcine cornea. Fibrillar structure is required for extracellular matrix integrity to avoid fatigue failure, a lack of which can lead to mechanical failure or tissue fibrosis^39^.

### Enzymatic degradation

Degradation of BPCDX, single-crosslinked BPC and the human cornea were evaluated following incubation in 1 mg ml^−1^ collagenase solution. BPCDX degraded more slowly than BPC, suggesting increased resistance of double crosslinking (Fig. 1e). Fifty percent weight loss took 18 h for BPC and 24 h for BPCDX, and 45 h for the human cornea, the assay thus being more aggressive than typical in vivo conditions. The human cornea initially gained weight (12–34 h) before rapidly losing weight, likely owing to endogenous crosslinks initially maintaining corneal integrity upon collagenase exposure; however, once most crosslinks were degraded by 12 h, the cornea absorbed water and swelled, resulting in weight increase, increased collagenase absorption and accelerated further enzymatic cleavage and degradation.

### In vitro cell biocompatibility

We previously reported cell compatibility of earlier versions of porcine collagen-based bioengineered materials^29–31^. In addition to third-party certified laboratory testing, we evaluated biocompatibility of BPCDX by seeding human corneal epithelial cells (HCE-2 cell line) on the surface of BPCDX. After 16 days of culture, live adherent cells of normal morphology were present on BPCDX at greater density than control wells (plastic), suggesting cell biocompatibility (Fig. 1f).

### End-stage UVC radiation sterilization

An independent GMP-certified laboratory confirmed BPCDX samples met a sterility assurance level (SAL) of less than one in one million units tested (1 × 10^−6^) under optimal UVC dosing. Further tests confirmed bioburden reduction from 4–50 CFU ml^−1^ for non-UVC-exposed samples to <1 CFU ml^−1^ for samples exposed to an optimum UVC dose. Mean bioburden reduction after UVC treatment was 22 CFU ml^−1^.

### Biological safety of BPCDX according to ISO 10993-1:2018

Biocompatibility testing protocols (Methods) were performed according to ISO 10993-1:2018 by an independent Good laboratory practice (GLP)-certified laboratory. Results indicated BPCDX is non-cytotoxic, non-irritating, non-toxic, non-sensitizing, non-genotoxic, non-pyrogenic and well-tolerated (reports not shown).

### Endotoxin test according to ISO 11979-08

Endotoxin is assessed during device manufacture as a routine quality control test according to ISO 11979-8 (‘Ophthalmic implants—Intraocular lenses-part 8: Fundamental requirements Amendment 1’) and ISO 15798. The acceptable endotoxin limit for ophthalmic devices as specified in ISO 15798 is 0.200 EU (endotoxin units) per device. Our in-house test results indicated a mean of <0.072 ± 0.036 (*n* = 4) EU per device. Independent laboratory test results indicated <0.091 ± 0.001 (*n* = 3) EU per device.

### Shelf-life stability tests according to ISO 11607

**Extended Data Fig. 1:**
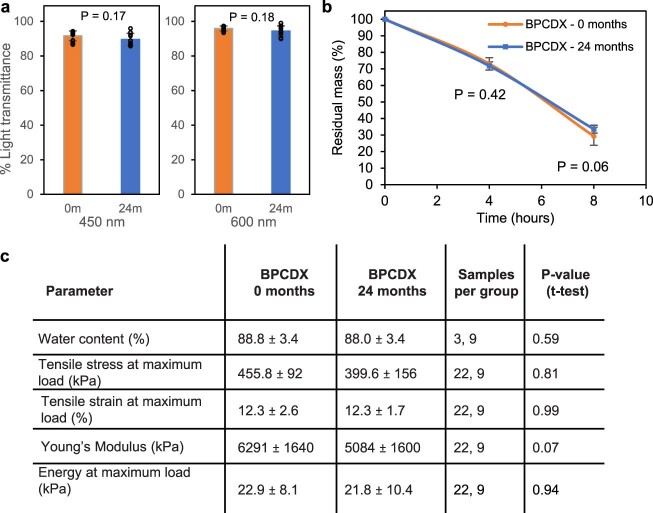
Real-time shelf-life stability test data for BPCDX after 24 months of storage at 7°C, relative to time zero samples. **(a)** Light transmission was maintained throughout the visible range of wavelengths (10 samples per group, BPCDX thickness 300 µm, diameter 9 mm, two-sided independent t-test). **(b)** Enzymatic degradation was unchanged, indicating implant integrity after long-term storage (9 samples per time point from 3 separate batches, BPCDX thickness 300 µm, diameter 9 mm, two-sided independent t-test). **(c)** Table indicating preservation of hydration and mechanical properties after long-term storage, with no significant differences relative to non-aged samples (550µm-thick ‘dog-bone’ specimens, two-sided independent t-test for each parameter). Data values in the graphs and table represent mean and standard deviation of measurements (error bars) from the indicated number of independent samples (at time 0 and 24 months, respectively). Source data

Real-time shelf-life stability was tested after storage of BPCDX devices at 7 °C for 24 months and accelerated shelf-life stability was tested after BPCDX incubation at 28 °C for 6 months. Test results from the real-time study (Extended Data Fig. 1) indicated maintained transparency, enzymatic resistance, water content and mechanical properties after 24 months of aging relative to non-aged control samples (*P* > 0.05 for all parameters). Third-party sterility testing of 24-month-aged samples by a GMP-certified accredited laboratory (MIKROLAB Stockholm AB) indicated the devices and packaging remained intact and fulfilled a SAL of no greater than 1:1 × 10^6^ units tested (reports not shown). Results indicate a minimum shelf-life stability of 2 years.

### In vivo biocompatibility, subcutaneous implantation in rats

Following subcutaneous in vivo implantation under the dorsal flank in three Wistar rats, no post-operative infection, wound abscess, delay in wound closure or suture-related complication was noted upon visual inspection (Supplementary Fig 1a). Histological analysis of excised tissue samples revealed intact BPCDX in close contact with surrounding host tissue (Supplementary Fig 1b), without apparent signs of degradation or thinning. Native host cells appeared at the BPCDX periphery but without apparent influx of inflammatory cells or new vessel growth. Low level expression of alpha smooth muscle actin (α-SMA) and type III collagen deposition were observed at the implant border (Supplementary Fig 1c,d).

### In vivo evaluation of BPCDX in a minipig keratoconus model

Ten Göttingen minipigs received femtosecond laser-enabled intrastromal keratoplasty (FLISK)^29,31^ surgery to first remove a 250-µm-thick, 7 mm diameter button of native stromal tissue in one eye, mimicking a thinned corneal stroma as in keratoconus (Supplementary Fig. 2). Thereafter, the removed native tissue was either replaced into the mid-stromal pocket in five minipigs (control autograft group), or a 280-µm-thick, 7-mm-diameter BPCDX was inserted in the remaining five minipigs. All operations were successful with the only intraoperative complication being variability in centration, with most implants being de-centered and skewed toward the limbus. This complication arises from the minipig anatomy, which prevents proper applanation of the eye owing to a backward rolling movement of the eyeball into the eye socket. The femtosecond laser is designed for the characteristics of human eyes.

**Fig. 2:**
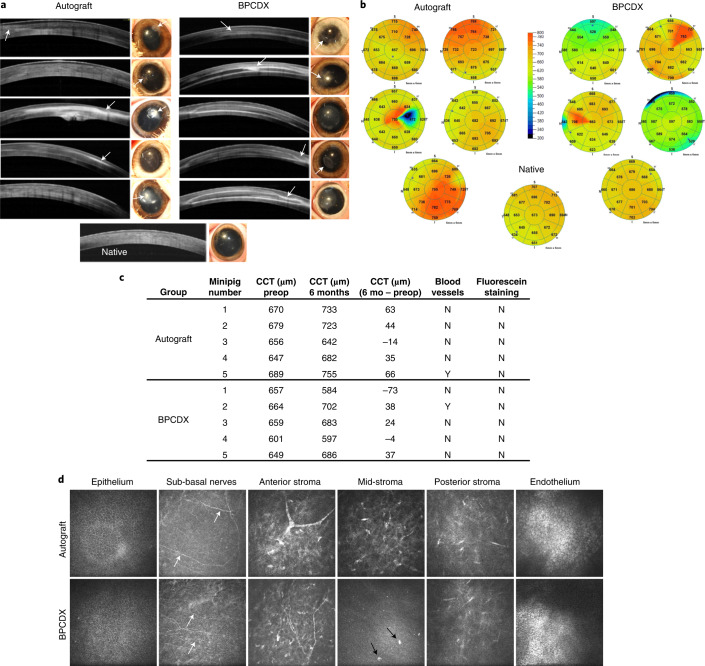
Results 6 months after intrastromal BPCDX implantation in minipigs. **a**, OCT images of the central 6 mm of cornea and corresponding photographs of operated eyes indicated localized thinning and loss of transparency in the access cut region in both groups (arrows in OCT scans and photographs). In the autograft group, one cornea (central image) exhibited loss of transparency, while another had partial loss of transparency (bottom image). In both cases, the implantation zone was skewed towards the limbus. The remaining three eyes were transparent with good thickness and only minor thinning at the access cut. In the BPCDX group, two eyes (second image from top, and bottom image) had a partially reduced transparency. In both cases, the implanted zone was skewed towards the limbus. In all eyes, transparency outside the access cut region was maintained. **b**, Pachymetry maps indicating corneal thickness 6 months post-operatively with color coding of thickness indicated by the grading scale, and mean thickness in µm indicated in each sector. BPCDX corneas exhibited similar thickness as the native porcine cornea. The native porcine cornea is shown for comparison purposes at the bottom of **a**,**b**. **c**, Table indicating pre-operative and post-operative mean corneal thickness and difference in corneal thickness in the central 2 mm zone as determined by OCT. Absence of positive fluorescein staining indicates complete epithelial wound healing. **d**, In vivo confocal microscopy images of porcine corneas at 6 months. In both groups, the epithelial cell mosaic appeared intact. Basal epithelium and sub-basal nerves (white arrows) were also observed, indicating preservation of the nerve plexus owing to minimal trauma during surgery. Anterior stromal nerves and keratocytes had normal morphology in both groups. The mid-stromal region appeared normal in autografts but BPCDX was devoid of keratocytes, except for individual cellular features (black arrows). Posterior stromal keratocytes and endothelial cells appeared normal and intact in both groups. All images in **d** are 400 × 400 µm^2^. Representative images are from five corneas per group with similar results obtained for each group. Source Data

Six months post-operatively, the central cornea was transparent in four of five autograft eyes and in five of five eyes with BPCDX. Microscopy and optical coherence tomography (OCT) imaging indicated partial thinning and reduced transparency in the access cut region containing the sutures (Fig. 2a), in both groups. Outside this region, thickness and transparency were maintained (Fig. 2a,b). OCT imaging indicated BPCDX stability without degradation and smooth native anterior and posterior refractive surfaces. Central corneal thickness was 657 ± 24 µm pre-operatively (Fig. 2c). After 6 months, central thickness was 650 ± 55 µm with BPCDX (*P* = 0.84 relative to pre-operative, 5 samples per group, paired *t*-test) and 707 ± 45 µm in autografts (*P* = 0.055, 5 samples per group, paired *t*-test). Change in thickness from pre-operative did not differ between groups (*P* = 0.45, *t*-test). Despite the de-centered implantation skewed towards the limbus, only one cornea in each group (BPCDX and autograft) had peripheral neovascularization.

In vivo confocal microscopy of all corneal layers 6 months post-operatively indicated an intact epithelial cell mosaic, preserved sub-basal nerves, and normal anterior and posterior stroma and endothelium in both groups (Fig. 2d). BPCDX remained free of cells apart from some cellular features. Intrastromal surgery preserved cellular layers, avoiding long-term deficits of nerves, epithelial cells and keratocytes seen in standard surgery^40,41^.

### Ex vivo analysis in the minipig keratoconus model

**Fig. 3:**
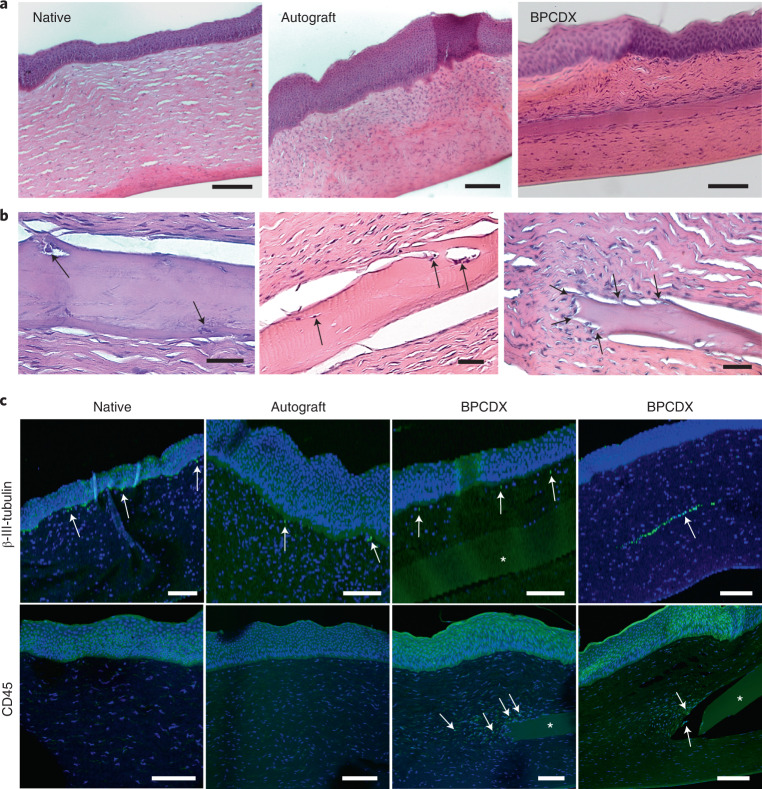
Postmortem histologic analysis of corneas in the minipig model. **a**, Hematoxylin and eosin (H&E) staining revealed autografts with thickened epithelium and increased presence of anterior stromal cells relative to the native porcine cornea. Epithelial and stromal layers in BPCDX corneas were uniform, with maintenance of overall corneal structure and anatomy. Representative images shown from three corneas per group. **b**, Three different BPCDX-implanted corneas where host cells (arrows, left and center images) migrated into the BPCDX. The edge of the BPCDX had multiple tissue attachments (arrows, right image) with cells appearing to migrate towards the BPCDX. **c**, Immunohistochemical analysis indicated sub-basal nerves by the β-III-tubulin marker (immediately below the epithelium, arrows), while a preserved stromal nerve in a BPCDX cornea was apparent. Leukocyte marker CD45 indicated weak staining of stromal cells located at the BPCDX periphery (arrows), suggesting leukocyte-mediated remodeling^43^ that differed in extent in different corneas (sections from two different BPCDX corneas shown). Leukocytes were absent in native and allograft corneas. All markers are indicated by green fluorescence, while a blue DAPI counterstain indicates the presence of cell nuclei. Non-specific diffuse signal from the green channel indicated the implanted BPCDX (asterisk in all panels). All images are representative images chosen from three corneas per group. Scale bars, 100 µm (**a**,**c**) and 50 µm (**b**). Source Data

Histology analysis indicated epithelial and stromal wound healing after intrastromal surgery (Fig. 3a). In autografts, epithelium was abnormally thick, and elevated presence of anterior stromal cells was noted. For BPCDX, epithelium exhibited normal epithelial stratification and implants were intact. Stromal cells aligned with BPCDX–host interfaces, with some cells inside the implant (Fig. 3b) but without implant degradation or strong inflammatory response. Tissue sectioning sometimes resulted in separation of the BPCDX from host tissue; however, regions of adherent tissue were still apparent (Fig. 3a,b). Host cells were present at the peripheral edges of the BPCDX where it appeared to integrate with host stroma. Host cell migration and stromal adhesion are suggestive of tissue biocompatibility of BPCDX. Immunostaining (Fig. 3c) revealed β-III-tubulin-positive nerves of the sub-basal nerve plexus, confirming in vivo observation of these nerves in all groups. Thicker stromal nerves were observed when a nerve path was present in the same plane as the corneal section. Sub-basal and stromal nerves are critical for epithelial health, stromal integrity and proper wound healing^42^. Leukocyte marker CD45 was absent in native stroma and autografts, while cells at the edge of the BPCDX were CD45^+^ to a variable degree, in line with previous observations of macrophage-related remodeling of the peripheral implant border^43^. Apart from these peripheral cells, no stromal inflammation was detected. Histopathologic findings in this model were similar to those seen after transplantation of human tissue^44^, with the exception that corneal nerves are typically absent following human corneal transplantation^40^.

### Minimally invasive BPCDX surgery in advanced keratoconus

Because of partial thinning and haze from suturing the access cut in the minipig model, we reverted to a suture-free implementation of FLISK^29,31^ with smaller access cuts to minimize complications in human subjects. Avoidance of sutures in FLISK removes additional barriers to implementation of the surgery in LMICs, circumventing issues of time, cost, suture-induced refractive errors and the need for additional hospital visits for subsequent suture adjustment and removal. In human subjects with advanced keratoconus but without scarring, we did not remove native corneal tissue and only added the BPCDX, simplifying the surgery to a single lamellar cut and access cut (Supplementary Fig. 3). Femtosecond laser-assisted surgery was used to ensure accuracy and reproducibility, but intrastromal pocket creation can be achieved manually without a laser^45,46^.

The pilot study sites have large populations with advanced keratoconus and severe visual impairment, who would not otherwise be treated owing to a lack of human donor tissue. Ethical approvals were obtained in Iran and in India to conduct a first pilot case series of BPCDX implantation, given the lack of treatment options. Surgeries were staggered as a precaution, to ensure proper assessment of initial post-operative response before treating further subjects. There were no deviations from the clinical trial protocol, including the pre-specified endpoints (see Methods for details of the protocol).

**Fig. 4:**
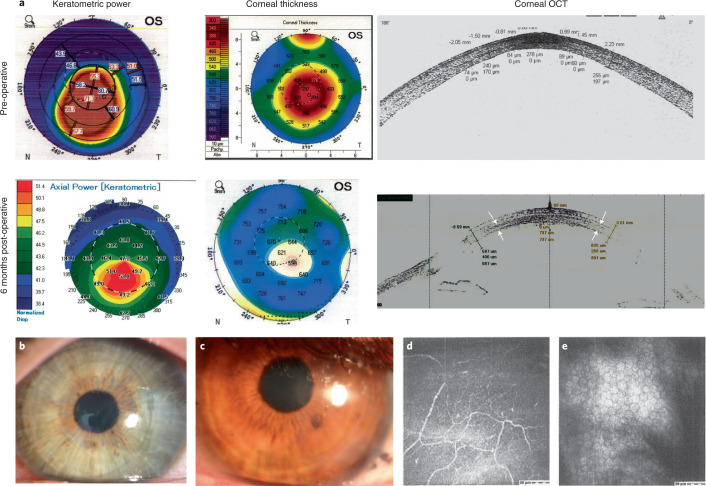
Clinical data from subjects in Iran receiving BPCDX. **a**, Keratometric and corneal thickness maps from the same subject indicate the thin and steep pre-operative cornea that was substantially thickened and flattened after intrastromal implantation of a 440-µm-thick BPCDX. The corresponding OCT cross-section scans indicate corneal thickness and shape before and after BPCDX implantation, with anterior and posterior borders of the BPCDX indicated by white arrows. The subject had an initial BSCVA of 20/200 that improved to 20/50 at 24 months post-operatively. **b**,**c**, Photographs of eyes from two subjects with the BPCDX four months post-operatively, indicating maintenance of corneal transparency. **d**,**e**, In vivo confocal microscopy images obtained from a single subject confirming the presence of sub-basal nerves (**d**) and endothelial cell mosaic (**c**) 6 months post-operatively. Endothelial cell density was 2,222 ± 62 cells per mm^2^ in the eye. As only this single subject was imaged by in vivo confocal microscopy, it is unknown if these images are representative. Images in **d**,**e** are 400 × 400 µm^2^.

**Fig. 5:**
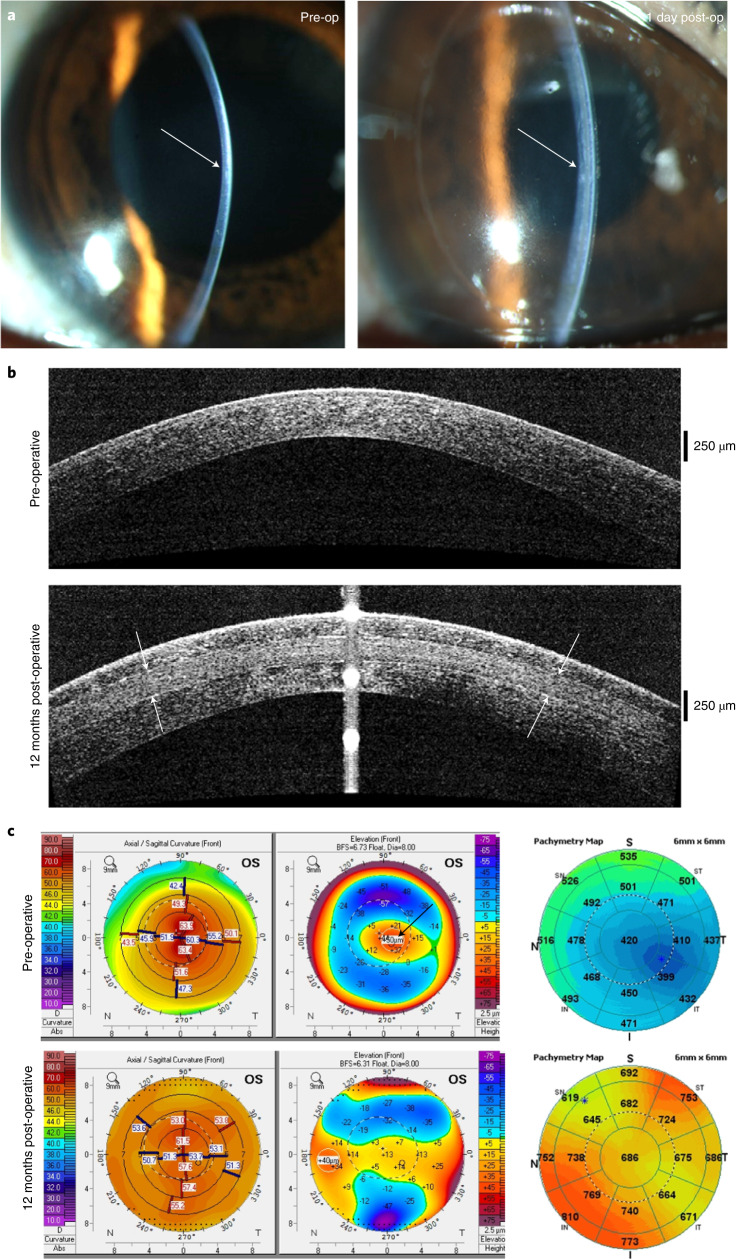
Clinical data from a subject in India receiving BPCDX. **a**, Slit-lamp photographs pre-operatively (left) and one day post-operative (right) with arrows indicating immediate change in thickness and curvature in the central cornea. **b**, OCT scans indicating sustained thickening and regularization of corneal curvature following implantation of 280-µm-thick BPCDX (anterior and posterior surfaces of BPCDX indicated by white arrows). **c**, Topographic maps (left, values given are keratometric power in diopters), anterior surface elevation maps (center, values are in µm displacement from a best-fit sphere) and OCT pachymetric maps (right, thickness in µm) from the same subject indicated substantial flattening of the steepest pre-operative central region (black arrow), and substantial increase in corneal thickness post-operatively. The subject initially had a best contact lens-corrected visual acuity (BCLVA) of 20/600. At 24 months BCLVA improved to 20/30.

We implanted the BPCDX into a laser-dissected intrastromal corneal pocket in 20 subjects without removal of host tissue. Post-operative slit-lamp biomicroscopy, Fourier-domain OCT (FD-OCT) and OCT pachymetry confirmed the intended placement of BPCDX and enabled assessment of transparency, stability and curvature (Figs. 4 and 5). The intrastromal procedure was feasible to implement in thin corneas without intraoperative complications. BPCDX was inserted into the intrastromal pocket with standard surgical forceps. A short 8-week post-operative medication regimen (Methods) was followed, during which no irritation or inflammation was noted. No extrusion or dislocation of BPCDX, and no thinning or scarring in the access cut region was noted in any subject. No conjunctival redness, abnormal anterior chamber angle or altered quality of the tear film was noted on slit-lamp observation. Slit-lamp photographs confirmed transparency (Figs. 4 and 5), and subjects examined with in vivo confocal microscopy exhibited intact sub-basal nerves and sufficient endothelial cell density (Fig. 4).

Safety and efficacy measures at the two-year post-operative follow-up are given in Table 1. Corneal transparency was maintained at the highest level (4+) post-operatively in all subjects, and no rejection, inflammation, vascularization, scarring or other adverse event occurred in any subject. In the Indian cohort, corneal transparency assessment during the first post-operative week revealed a transient haze in 5 of 8 subjects, reducing the transparency grade to 3+. At the 1-week follow-up, transparency increased to 4+ and remained stable thereafter in all subjects. OCT scans indicated similar light scatter in native corneal tissue and BPCDX, and pachymetry indicated sustained thickening and flattening of initially steep corneas. Intraocular pressure was measured in Indian subjects, and a small increase was noted but was not considered high and did not require pressure-lowering medication. In terms of efficacy, central corneal thickness increased significantly by several hundred microns in all subjects (*P* < 0.001), which was maintained at 24 months. Mean anterior corneal curvature (*K*_m_) and maximal corneal apical curvature (*K*_max_) were both significantly reduced in both cohorts (*P* = 0.002 and 0.01, respectively), indicating effective flattening. Normalization of corneal curvature resulted in improved best spectacle-corrected (BSCVA; Iran, *P* < 0.001) and best contact lens-corrected visual acuity (BCLVA; India, *P* < 0.001; Table 1 and Supplementary Table 2). Although no specific measures were taken to optimize post-operative refractive outcome, 11 of 12 subjects in the Iranian cohort and all 8 subjects in the Indian cohort experienced substantial gains in visual acuity, with a final corrected visual acuity of 20/58 in Iranian subjects and a remarkable 20/26 in Indian subjects. All subjects in both cohorts were contact lens intolerant pre-operatively but all could tolerate contact lens wear for extended periods at 24 months post-operative. Finally, while a total 14 of 20 subjects were legally blind pre-operatively (BCVA of logMAR ≥ 1.30 and contact lens intolerant), none were blind in the operated eye at final follow-up.

**Table 1:** 24-month clinical safety and efficacy measures of BPCDX implantation in advanced keratoconus patients in two pilot clinical case series

Iranian cohort	Pre-operative	24 months	*P* value^1^
Number of subjects	12	12	
Subject Age (years)	30.8 ± 9.7		
Age range (years)	18–43		
Number of females/males	5/7		
Number of 280/440 µm BPCDX	5/7		
Corneal transparency	Good (4+)	Good (4+)	
Central corneal thickness (µm)	446 ± 66	728 ± 88	2.4 × 10^−6^
Thinnest point (µm)	405 ± 69	683 ± 88	1.8 × 10^−5^
Corneal volume (mm^3^)	57.1 ± 3.0	75.2 ± 7.4	2.0 × 10^−5^
Mean keratometry *K*_m_ (D)	57.7 ± 6.5	52.8 ± 1.9	0.013
Max keratometry *K*_max_ (D)	69.1 ± 9.0	58.8 ± 4.0	0.002
BSCVA (logMAR)	1.22 ± 0.54	0.46 ± 0.22	2.5 × 10^−4^
logMAR lines gained, BSCVA		7.6 ± 6.1	
Contact lens tolerant subjects	0/12	12/12	
Legally blind subjects^2^	6	0	
**Indian Cohort**	**Pre-operative**	**24 months**	***P*** **value**^1^
Number of subjects	8	8	
Subject Age (years)	26.6 ± 4.7		
Age range (years)	19–31		
Number of females/males	2/6		
Number of 280/440 µm BPCDX	8/0		
Corneal transparency	Good (4+)	Good (4+)	
Central corneal thickness (µm)	378 ± 15	587 ± 18	7.0 × 10^−9^
Mean keratometry *K*_m_ (D)	57.1 ± 7.0	48.4 ± 1.5	0.011
Max keratometry *K*_max_ (D)	62.7 ± 7.5	48.8 ± 1.3	0.002
BCLVA (logMAR)	1.63 ± 0.15	0.12 ± 0.11	1.2 × 10^−7^
logMAR lines gained, BCLVA		15.1 ± 2.0	
Intraocular pressure (mmHg)	11.8 ± 0.7	13.5 ± 1.5	0.009
Contact lens tolerant subjects	0/8	8/8	
Legally blind subjects^2^	8	0	

^1^Final follow-up relative to pre-operative, two-tailed paired *t*-test.

^2^Legally blind if logMAR BCVA ≥ 1.30 and contact lens intolerant.

Source data

## Discussion

Our results provide evidence that intrastromal implantation of a cell-free bioengineered collagen-based material can be a safe and feasible means to reverse the pathologic corneal thinning and deformation in advanced keratoconus. Following BPCDX implantation, transparency was maintained without degradation, scar formation, adverse reactions or events requiring hospitalization, intensive therapy or further surgical intervention—thus meeting safety criteria. The procedure achieved an increase in central corneal thickness of 285 ± 99 µm in the Iranian and 209 ± 18 µm in the Indian cohort at 2 years post-operative, also flattening the excessively steep cornea with a mean decrease in *K*_max_ of 11.2 ± 8.9 D in the Iranian and 13.9 ± 7.9 D in the Indian cohort, meeting efficacy criteria in 90% (18/20) of cases, while vision gain was achieved in 95% (19/20) of cases at 24 months. We expect these gains to be stable, on the basis of our long-term experience with a single-crosslinked collagen-based bioengineered implant, where after initial thinning of approximately 180 µm, implanted biomaterials remained stable for 4 years^28^ and in a later follow-up, stability was confirmed at 8 years (unpublished data). The BPCDX is expected to have added stability relative to the previous material, on the basis of material optimization (Supplementary Table 1), which in this study led to sustained corneal thickening with the absence of post-operative thinning. The evidence is suggestive that BPCDX would be at least as stable in the long term as previous, softer implanted materials.

The visual gains we report represent equivalent outcomes to historical results of standard penetrating corneal transplantation surgery for keratoconus with human donor corneas (Supplementary Table 3). The historical dominance of DALK and PK have until now remained unchallenged (notwithstanding the lack of donor tissue) because of the unmatched visual acuity attainable by these standard techniques. With individualized attention to corneal thickness, curvature and refractive errors, our results thus far suggest that final visual acuity following BPCDX implantation could possibly exceed DALK or PK outcomes; future clinical studies will be needed to test this assertion. Notably, vision in the present study at 24 months was either equivalent (Iranian cohort) or improved (Indian cohort) relative to an earlier biosynthetic collagen-based material implanted by more invasive lamellar keratoplasty in ten Swedish subjects^27^. In that study, however, the mean long-term *K*_max_ decrease was a non-significant 2.5 D, and subjects had a milder grade of initial keratoconus^47^ with substantially less steep corneas and less advanced disease relative to the subjects in the present cohorts (Supplementary Table 3).

Topical corticosteroids are typically applied for up to one year following DALK and PK^48,49^ to prevent suture-induced inflammation and neovascularization, and additionally the techniques require multiple post-operative visits and corrective surgical procedures^50–52^ for re-suturing, suture removal and to manage suture-related complications and post-operative astigmatism caused by suture-induced tissue deformation and donor–recipient tissue mismatch^20^. Our intrastromal technique avoids suture-related issues and visits, required only 8 weeks of immunosuppression, and use of a single 2-mm incision in the Indian cohort is half the incision size of typical intrastromal incision^21^ and preserves corneal epithelium and sub-basal nerves to a similar level as in minimally invasive cataract surgery. Previous intrastromal procedures using donor lenticules are limited to a maximum corneal thickness increase of 100 µm, carry the risk of disease transmission and donor tissue rejection, require rapid transplantation owing to short lenticule storage times, and need customized surgical tools^21^. For these reasons, the present intrastromal technique can be advantageous from a safety and feasibility perspective, regardless of bioengineered tissue use.

Our adaptation of the intrastromal procedure has not been described previously in humans to our knowledge. It differs from standard thin lenticule implantation by use of implants an order of magnitude thicker (280–440 µm) inserted through a minimal 2-mm incision, with the effect of reshaping both the central and peripheral cornea to provide significant flattening of the initially steep corneal curvature to a degree not yet shown to be possible using human donor tissue implantation. Ganesh and colleagues^33^ used thin annular-shaped human donor lenticules to treat keratoconus, thickening the central stroma by <20 µm to achieve 2–4 D of flattening after 6 months. Later studies used circular disks of donor tissue thickening the central stroma by 50 µm to achieve 5 D of flattening after 6 months^21^, or a central thickening of 30 µm to achieve 3 D of flattening after 3 years^34^. These previous studies reported vision improvement of 2–3 logMAR lines of best-corrected acuity. Our surgical approach achieved an unprecedented central corneal thickness increase ten times that of previous studies sustained for at least 2 years, resulting in three times the degree of corneal flattening of previous studies and a substantially greater improvement in vision.

Before operations for advanced keratoconus, patients are intolerant to wearing contact lens for long periods because the curvature of the cornea is excessively steep and thus contact lenses do not properly sit on the corneal surface, leading to lens movement, refractive irregularities and discomfort or irritation in the eye. Post-operatively, the high degree of flattening achieved in the present cohorts resulted in better fitting of contact lenses and therefore tolerance for extended periods in all subjects without discomfort. After standard PK, patients can become tolerant to contact lens wear for varying periods (2–12 h per day), in about 50% of cases^53^. In the relatively small cohorts in this study, long-term contact lens tolerance was noted post-operatively in 100% of subjects, likely resulting from the significant corneal flattening achieved following BPCDX implantation.

While previous clinical studies focus on human donor tissue, bioengineering implantable tissue is the key to addressing the global burden of corneal blindness. A growing number of bioengineered materials are being evaluated in animal models, but to date only a few have reached human studies. A summary of recent clinical and preclinical studies of corneal bioengineering technologies is given in Supplementary Table 4. Most clinical studies, while having achieved significant advances in biomaterial properties, have addressed relatively rare causes of corneal blindness such as chemical burns^54,55^, infections^55–58^, ulcers^59^ or high-risk cases^54,55^ with the goal to stabilize the condition and avoid blindness, but not to optimize vision. Only two clinical studies to date have addressed keratoconus, a condition that impairs millions globally, aiming to provide vision gains comparable to standard PK or DALK. One approach, while innovative and promising, still requires human donor tissue, tissue banking and additional liposuction surgery^34^, and has to date provided limited corneal thickening, flattening and BCVA gain. The other study, conducted by several of the present authors^27,28^, failed to thicken the cornea or provide complete transparency, with the biomaterial unable to withstand enzymatic degradation. Moreover, the implants in that study were not packaged, tested for shelf life or full ISO compliance, and surgery led to suture-related complications, scarring and an uneven corneal surface.

BPCDX is the only technology to date combining a chemical and photochemical process for double crosslinking to stabilize collagen in a viscous form that avoids a high concentration of harsh crosslinkers, minimizing cytotoxicity. The high-purity raw collagen, vacuum evaporation process achieving high collagen content, increased chemical-crosslinker-to-collagen ratio and optimized photochemical crosslinking, along with other key biomaterial properties, significantly differentiates the BPCDX from previous materials tested in humans (see Supplementary Table 1 for a detailed comparison). These properties impart a stability to the material after human implantation that was not observed in a previous clinical study where the implanted biomaterial became thinner post-operatively^27,28^. Additionally, no previous study has, to our knowledge, demonstrated a commercially and clinically viable, GMP-grade cornea bioengineered from a sustainably sourced, cost-effective, widely available and FDA-approved raw material. No other technology has achieved manufacturability, packaging, sterility and long shelf life that are ISO-compliant and independently third-party certified. Yet these often-overlooked aspects are critical for addressing the global lack of donor cornea tissue in practical terms. From a safety perspective, BPCDX did not result in thinning, loss of transparency, neovascularization, rejection or other adverse event observed to varying degrees in most preclinical and clinical studies to date. From an efficacy perspective, no previous study has, to our knowledge, achieved full corneal transparency in vivo with sufficient corneal thickening and flattening, or with significant visual acuity gains as reported here; at best modest vision gains have been achieved in previous studies, although with post-operative complications^27,28^ or with the use of human donor tissue^21,22,33,34^.

BPCDX is mechanically more robust than earlier versions we previously developed^29,30,43^, with superior toughness, Young’s modulus and stress tolerance translating into an ability to withstand surgical implantation, mechanical forces and enzymes in vivo. Our results suggest it may not be necessary for bioengineered tissue to match all the mechanical properties of the native cornea to be therapeutically effective. Standard corneal transplantation with mechanically tough human donor tissue often results in scar tissue formation at host-to-implant interfaces^50,60^, a phenomenon we did not observe with the mechanically softer BPCDX. While still not achieving the tensile strength of the normal human cornea as assessed in laboratory tests, BPCDX nonetheless exhibited resistance to degradation in vivo to maintain its integrity and sustain corneal thickness for at least 2 years after human implantation. The material had adequate stiffness to reshape the cornea and normalize curvature by mechanically pushing outwards the anterior and posterior recipient stromal layers and withstanding the intraocular pressure, which was maintained at normal levels following implantation.

We believe it is important to report results from this pilot study in LMICs for several reasons. Although recruitment continues, initial safety and efficacy are sufficiently positive to motivate prospective, randomized controlled trials to compare to standard DALK or PK. The reported results have been submitted to regulators and regulatory approval has been granted to conduct a clinical trial in the European Union for presbyopia using the BPCDX and the intrastromal implantation method (Clinicaltrials.gov: NCT04465409). Finally, we envision that surgeons could adopt the intrastromal procedure as a simpler, safer and less invasive option for keratoconus than standard DALK or PK, even with donor corneas; trials to this effect are warranted.

On the basis of our validated device manufacturing, packaging and shelf-life stability results, BPCDX could be widely distributed and stored up to 2 years before use, providing an alternative to the paradigm of securing consent for donation, harvesting corneas postmortem, pathogen testing and short-term storage in accredited facilities (to a maximum of 7 days). BPCDX can be stored at room temperature or in a refrigerator without special medium or control procedures required to ensure cell viability in human donor tissue (or absence of cells and DNA^8^ in decellularized material). As BPCDX is packaged and sterile, it does not require pathogen testing needed for human tissue, thus representing a safer alternative in times of viral outbreaks. The intrastromal procedure we describe—a laser-assisted or manual mid-stromal lamellar dissection to create an intrastromal pocket in the recipient cornea—would not require extensive training and could be easier and faster for surgeons to implement, given that the procedure is suture-free. Previous clinical studies have demonstrated reproducible manual stromal dissection^22,23^; a surgical laser is thus not a requirement. Our results indicate the potential to restore vision without long-term follow-up or additional procedures and could be applied in large patient populations to achieve even a modest vision gain. Crossing the threshold from severe visual impairment to low or moderate vision can improve quality of life even where resources may not allow for post-operative hospital visits or a long post-operative medication regime.

Our pilot studies had several limitations. Sites lacked control subjects receiving donor corneas, precluding direct comparison to standard surgery for advanced keratoconus. Biases towards subject inclusion may have existed, given the lack of treatment alternatives. Studies were performed independently, with patient selection, surgical parameters, selection of BPCDX size and thickness, post-operative medications, clinical data collection and measurement of outcome parameters dictated by the local investigators, preventing a combined analysis of outcomes. Also, procedures were not optimized for maximum vision gain. Differences in vision improvement in subjects may have resulted from the thicker BPCDX (440 µm; Table 1) used in some subjects in Iran (chosen as a safety measure against possible thinning) and may also be related to poorer pre-operative vision, thinner corneas and less steep maximal keratometry in the Indian cohort, resulting in greater vision gains. Although only manufactured in two thicknesses for pilot studies, BPCDX can be manufactured for customized treatment, including non-uniform or tapered thickness for optimization of refraction and visual outcome. Future studies should define pre-operative corneal parameters and BPCDX designs resulting in optimal vision outcomes. Also, tissue integration, stability and clinical outcomes should be investigated in the longer term. Quantitative assessment of anterior chamber angle, conjunctival redness, ocular surface and tear film properties, and the posterior segment is needed to discern possible effects of intrastromal BPCDX implantation on other ocular structures. Generalizability of the results to broader populations (such as those with stromal scarring, by first removing tissue as in the minipig model) and to different centers requires further investigation.

Given the initial safety and efficacy outcomes and potential for patient benefit relative to the risk of adverse events and given the shortage of donor tissue, the present results motivate the need for randomized, controlled studies.

## Methods

### Manufacturing of collagen-based corneal stromal equivalent

We developed a collagen-based corneal stromal substitute, BPCDX. BPCDX was manufactured following principles of good manufacturing practices (GMP), in a Class 5 (according to ISO-14644-1) air quality laminar flow clean-room facility in Linköping, Sweden by LinkoCare Life Sciences AB. Although the BPCDX is subject to an end-stage sterilization process, substantial efforts are made throughout the manufacturing process to maintain raw materials, intermediate products and the final product as aseptic as possible.

In brief, the fabrication procedure was as follows. Medical-grade purified freeze-dried type I porcine dermal atelocollagen was purchased from SE Eng Company (South Korea). The collagen was dissolved in PBS at room temperature to form a 5% collagen solution. The collagen solution was then exposed to a controlled vacuum evaporation^29^, and crosslinkers 1-[3-(dimethylamino) propyl]-3-ethylcarbodiimide methiodide (EDCM), *N*-hydroxysuccinimide (NHS) and riboflavin (vitamin B2) (Care Group, Vadodara) were added at 1% (w/v) ratios. The solution was mixed thoroughly and dispensed into custom curved contact lens molds with spacers of 280 µm and 440 µm used to delineate the final device thickness. Molds were clamped and samples were cured at room temperature. Removal from molds was achieved by immersion in PBS for 1 h at room temperature. Finally, BPCDX was rinsed with sterile PBS to extract any reaction residues. Following this chemical crosslinking, a second photochemical crosslinking of samples was performed by exposure to ultraviolet A (UVA) light, following the protocol of UVA–riboflavin corneal collagen crosslinking commonly used clinically^32^. We employed this second photochemical crosslinking step aiming to further improve the BPCDX mechanical strength, resistance to degradation and long-term stability. Notably, the BPCDX differs from single-crosslinked recombinant human-collagen-based implants evaluated previously in humans^27,28,62^ in several important ways. Firstly, we used medical-grade and FDA-approved porcine dermal collagen as a starting material, the ultra-purity of which results in a more mechanically robust hydrogel and reproducible batch-to-batch mechanical properties. The use of a novel vacuum evaporation technique enables a high collagen content (12–18%) to be controllably achieved^29^, relative to previous human-tested implants limited to 10% collagen by weight (the native human cornea is 13.6% collagen)^27^. We additionally increased the ratio of chemical crosslinkers to collagen from 0.4:1 to 1:1 to achieve a higher degree of crosslinking in comparison to earlier implants. Further, our use of the crosslinker EDCM^29^ (as opposed to EDC^27^) and subsequent UVA–riboflavin crosslinking with optimized dose and exposure parameters, differentiates the present BPCDX from previous collagen-based hydrogels, imparting additional strength and resistance to degradation (see Supplementary Table 1 for full comparison of manufacturing parameters and material properties).

### Packaging of BPCDX

After manufacture, BPCDX is extracted from any reaction residues and rinsed thoroughly with sterile phosphate-buffered saline (PBS) in class 5 (Class 1000) laminar flow hoods and stored in sterile PBS in a sterilized, sealed blister-packed container as shown in Supplementary Fig. 4a. Primary blister packs are labeled according to ISO 15223-1:2012 and EN 1041:2008 as shown in Supplementary Fig. 4b. The entire blister cup package and instructions for use are inserted into a small outer packaging box for protection during transportation and storage (Supplementary Fig. 4c).

### Sterilization of BPCDX

To provide an additional level of device safety beyond an aseptic manufacturing process, we implemented an additional end-stage sterilization procedure. Conventional sterilization techniques (for example, dry heat, steam, ethylene oxide, gamma irradiation and electron beam irradiation) widely used for rigid medical device sterilization have not been tested or validated for soft, tissue-engineered devices such as hydrogels, where they are likely to result in denaturation and loss of device integrity. For this reason, we developed a sterilization procedure using a pulsed UVC irradiation system (Xenon Z-1000 Flash UV Lamp System). Pulsed UV light is known for its ability to inactivate microbes, is widely used in the food industry and is gaining popularity as a sterilization method^63,64^.

Sterilization validation of BPCDX implants was performed according to ISO 14937:2016 and ISO 11137-1-3:2017 sterilization standards. Two sets of product samples were exposed to pulsed UVC light at two sterilization doses separately, and then tested for key properties and sterility to investigate if the UVC exposure at these dosages impacted BPCDX properties and/or packaging. Sterility tests were both performed internally using tryptic soy broth sterility test method and externally by an ISO-certified microbiology lab (MIKROLAB Stockholm AB) for independent sterility and bioburden tests.

Quality control of samples and their packaging was conducted by visual inspection, mechanical, optical, water content, analysis of physical dimensions and collagenase degradation tests on the UVC**-**exposed samples and non-sterilized samples as controls. Energy intensity of the pulsed UVC light at all positions of the sterilization tray was measured using a UVC Nova II Laser Power/Energy Meter (Ophir Spiricon Europe GmbH) and a LiteMark-XL light intensity monitor.

### Optical transparency

Light transmission through BPCDX was measured across the UV and visible light spectrum (200–700 nm) at room temperature, using a High Performance USB4000 UV-Vis Spectrophotometer (Mettler Toledo). To enable direct comparison with light transmission through the native human cornea, BPCDX samples were 550 µm thick. Samples were immersed in PBS during measurement, and light transmission was compared with published data from healthy human corneal tissue^35^. Measurements were made for three independent samples per spectrum.

### Mechanical properties of scaffolds

Tensile strength, elongation at break (elasticity), elastic modulus (stiffness) and energy at break (toughness) of BPCDX were measured with an Instron Automated Materials Testing System (Model 5943 Single Column Table Frame) equipped with BlueHill software (v.3), a load cell of 50 N capacity and pneumatic metal grips at a crosshead speed of 10 mm min^−1^. Test specimens were made by molding and curing the samples into dumbbell-shaped Teflon molds followed by equilibration in PBS. Specimens were attached to the grips and tensile force was applied until the sample break point. Data were automatically recorded by the software, and 22 dumbbell-shaped samples were used for each mechanical test.

### Characterization by scanning electron microscopy

Morphology of BPCDX and the native porcine cornea was investigated using a Zeiss SEM (LEO 1550 Gemini). PBS-equilibrated samples were washed in water, frozen overnight at −80°C and lyophilized for 12 h. Samples were cut and attached onto metal holders using conductive double-sided tape, and sputter coated with a gold layer for 60 s at 0.1 bar vacuum pressure (Cressington Sputter Coater, 108) before SEM examination. SEM micrographs were taken at various magnifications at 25 kV and 5 kV for porcine cornea and BPCDX samples, respectively.

### Enzymatic degradation test

Test samples of BPCDX, a version (BPC) that was singly crosslinked with EDC-NHS^29^ and donor human cornea, all with a thickness of 500 µm, were exposed to collagenase Type I (from *Clostridium histolyticum*) and their residual mass percentage as a function of time was measured. Trizma base (Tris base), 2-amino-2-(hydroxymethyl)-1,3-propanediol was used for preparing Tris-HCl buffer. Test samples were equilibrated in 5 ml 0.1 M Tris-HCl buffer (pH 7.4) containing 5 mM CaCl_2_ at 37 °C for 1 h. Subsequently, 1 mg ml^−1^ (288 U ml^−1^) collagenase solution was added to give a final collagenase concentration of 5 U ml^−1^ (17 µg ml^−1^). The solution was replaced every 8 h to retain sufficient collagenase activity. Samples were weighed at various time intervals after gently blotting away the surface water. Three replicates of each sample type were tested. The percent residual mass of the sample was calculated according to the ratio of initial sample weight to the weight at each time point.

### In vitro cell biocompatibility

Culture of human corneal epithelial cells (HCE-2 50.B1 cell line, lot no. 70015331, ATCC) was established according to the manufacturer’s instructions. In brief, serum-free keratinocyte growth medium (1×, Gibco) was supplemented l-Glutamine, 5 ng ml^−1^ epidermal growth factor and 0.05 mg ml^−1^ bovine pituitary extract (BPE), 500 ng ml^−1^ hydrocortisone and 0.005 mg ml^−1^ insulin (Gibco). A T-75 cell culture flask was pre-coated with a mixture of 0.01 mg ml^−1^ fibronectin, 0.03 mg ml^−1^ bovine collagen type 1 and 0.01 mg ml^−1^ bovine serum albumin (BSA), and was incubated overnight at 37 °C. The next day, the excess coating was aspirated, and the flask was allowed to stand for 15 min before seeding the cells. The HCE-2 cell vial was thawed, and cells were seeded at a density of 10^4^ cells per cm^2^ on the pre-coated flask. Cells were incubated at 37 °C at 5% CO_2_, and growth medium was changed every other day.

BPCDX (300 µm thick) pre-cut to 8 mm diameter were rinsed in PBS and equilibrated in the complete keratinocyte serum-free medium for 2 h in a humidified cell culture incubator. BPCDX samples were then laid down (concave side down) onto the bottom of a 48-well cell culture plate and allowed to adhere to the bottom of the plate for 2 h in an incubator at 37 °C. Three wells were used for BPCDX samples, and three were used as controls (that is, no biomaterial attached to the bottom of the well).

Upon confluence of the seeded HCE-2 cells in the culture flask, the cells were trypsinized with Trypsin-EDTA, then trypsinization was stopped with a complete growth medium and cells were harvested, counted and seeded into the six prepared wells. Cells were seeded at a density of 10^5^ cells per well for the control and BPCDX-covered wells and incubated for 1 h before adding additional medium to reach a final volume of 200 µl growth medium per well. Growth medium was changed every other day, and cells were maintained in culture for 16 days. On day 16, the cells were washed and covered with fresh medium. Cells were stained with NucBlue Live cell stain (Hoechst 33342, Thermo Fisher Scientific) according to the manufacturer’s instructions. Images of stained cells were captured using a Leica DMi8 inverted live-cell microscope under ultraviolet light excitation (385 nm) to detect live cells with fluorescent blue nuclear stain. Brightfield images were additionally obtained to observe cell morphology.

### Biological evaluation according to ISO 10993-1:2018

Biocompatibility testing of the BPCDX was performed in conformance to GLPs as per US FDA CFR Title 21 Part 58 and according to ISO 10993 and ISO 11979 standards by an external GLP-certified contract research organization (BioNeeds Private Limited, India). BPCDX underwent the following in vitro and in vivo biocompatibility studies:

1. ISO 10993-3: genotoxicity, carcinogenicity and reproductive toxicity (bacterial reverse mutation tests (ref. report no. BIO-GT-348))
2. ISO 10993-3: genotoxicity, carcinogenicity and reproductive toxicity (in vitro mammalian chromosome aberration test (ref. report no. BIO-GT-349))
3. ISO 10993-3: genotoxicity Ames Test (ref. report no. BIO-GT-358)
4. ISO 10993-3: genotoxicity, carcinogenicity and reproductive toxicity (mammalian erythrocyte micronucleus test in Swiss albino mice) (ref. report no. BIO-GT-350)
5. ISO 10993-4: in vitro hemolysis test (ref. report no. BIO-TX-1575)
6. ISO 10993-5: in vitro cytotoxicity:
  - In vitro cytotoxicity (direct contact method) (ref. report no. BIO-GT-346)
  - In vitro cytotoxicity (elution method) (ref. report no. BIO-GT-347)
7. ISO 10993-10: skin sensitization in guinea pigs (ref. report no. BIO-TX-1574)
8. ISO 10993-10: in vivo ocular irritation in rabbits (polar + non-polar) (ref. report no. BIO-TX 1584)
9. ISO 10993-11: acute systemic toxicity in Swiss albino mice (ref. report no. BIO-TX-1576)

### Bacterial endotoxin test according to ISO 11979-08

To ensure the sterile BPCDX would be safe for human use, endotoxin testing was performed internally and by an independent laboratory (S2 Medical AB) using an Endosafe-PTS endotoxin analyzer (Charles River) with a rapid, point-of-use spectrophotometer and USP/BET-compliant disposable cartridges for real-time endotoxin testing. The limulus amebocyte lysate cartridges used in the tests are FDA-licensed for in-process and final product release testing, ensuring regulatory compliance of the output results. In brief, the tests were performed and reported according to ISO 11979-8 (ophthalmic implants-intraocular lenses-part 8: fundamental requirements amendment 1) and ISO 15798. The acceptable endotoxin limit for ophthalmic devices as specified in ISO 15798 is 0.2 EU per device against which BPCDX test results were evaluated.

### Shelf-life stability tests according to ISO 11607

Verification of stability is a time-consuming and resource-intensive task in the development of new medical devices and is often overlooked in research studies. For wide distribution of the BPCDX to regions with the greatest unmet need, shelf-life studies are critical to ensure the device produced in a normal manufacturing process functions as intended despite logistical, storage and other barriers that may occur in the distribution chain. Often devices are tested under accelerated conditions to increase the rate of chemical and/or physical degradation and therefore decrease the time required to obtain stability data. Long-term or real-time studies are still needed however, as the accelerated and long-term results may differ. For packaged and sterilized BPCDX, we therefore performed an accelerated shelf-life stability study by incubating devices at 28 °C for 6 months, and we performed a real-time shelf-life stability study by incubating devices at 7 °C for 2 years. Control BPCDX samples not subjected to aging (time-zero samples) were prepared and tested for visual appearance, mechanical properties, optical transmission, water content, size, collagenase degradation, in-house tryptic soy broth sterility test and for sterility tests conducted by an independent GMP-certified laboratory (MIKROLAB Stockholm AB). The accelerated and real-time aged samples were also tested for the above properties and compared with the control samples.

### In vivo biocompatibility, subcutaneous implantation in rats

To test compatibility of BPCDX after surgical implantation in vivo, we used a model of subcutaneous implantation in rats as described previously^43^. Three male Wistar rats aged 8 weeks were given a pre-operative analgesic (0.01 mg buprenorphine) by intraperitoneal injection the day before surgery, day of surgery, and 1 and 2 days after surgery. Under general anesthesia (25 mg ml^−1^ ketamine and 0.5 mg ml^−1^ dexmedetomidine hydrochloride), a 2-cm long paravertebral cutaneous incision was made into the dorsal flank of the rat, after which a subcutaneous pocket was created by blunt dissection. A 1-cm square piece of the BPCDX was inserted into the pocket, and the pocket was sealed with three absorbable 9-0 Vicryl sutures. Eight weeks post-implantation, rats were euthanized, and the tissue region surrounding the implant zone was excised and prepared for immunohistochemical analysis. The procedures were performed after obtaining ethical approval from the Linköping Animal Ethical Review Board (permit ID 585), with procedures in compliance with EU Directive 2010/63/EU on the protection of animals used for scientific purposes.

### Minimally invasive BPCDX implantation in minipigs

To evaluate implantation of BPCDX in vivo in the cornea, we designed a model of keratoconus in the Göttingen minipig to create an artificially thin native corneal stroma mimicking the pathologic thinning in advanced keratoconus. To achieve this, we used an ophthalmic femtosecond laser (iFS 150, Abbott Medical Optics) and modified the technique we previously reported in rabbit models, termed FLISK^29,31^. Animal experiments were performed after receiving approval from the Linköping Animal Ethical Review Board (permit ID numbers 153 and 37–16) and adhered to the guidelines of the ARVO Statement for the Use of Animals in Ophthalmic and Visual Research and the Directive 2010/63/EU. All surgeries were performed in a licensed large-animal surgical suite with controlled temperature and humidity at the Linköping University Translational Medicine Center, under supervision of the university veterinarian and animal care team. Ten female Göttingen minipigs (Ellegaard Göttingen Minipigs A/S) aged 6 months and weighing 15 kg were pre-medicated with sedatives (3 mg kg^−1^ tiletamine HCl + 3 mg kg^−1^ zolazepam HCl, Zoletil and 0.06 mg kg^−1^ medetomidine HCl, Dexdomitor), administered intramuscularly. Anesthesia was initiated with 0.2 mg kg^−1^ Propofol, administered intravenously through a venous catheter placed in the ear vein. Thereafter, the animals were intubated with anesthesia maintained using 0.5–3.0% isoflurane gas. During anesthesia, hydration was maintained by intravenous administration of Ringer’s acetate solution at 10 ml kg^−1^ h^−1^. Immediately before surgery 0.02 mg kg^−1^ atropine was given by intramuscular injection. Topical anesthesia (0.5% tetracaine HCl eye drops) was given before laser surgery, and all surgeries were performed in a single eye per animal.

To achieve a thinner stroma mimicking advanced keratoconus, we used the femtosecond laser to cut a central mid-stromal button in the cornea 7 mm in diameter and 250 µm in thickness, with the anterior surface of the button located 200 µm below the corneal surface (Supplementary Fig. 2). To achieve this we pre-programmed the laser to perform cuts in the following order: a posterior circular lamellar planar cut of 7.1 mm diameter located 450 µm below the corneal surface, a 360° circular side cut of 7.0 mm diameter extending from the posterior lamellar plane 250 µm anteriorly, and an anterior circular lamellar plane of 7.1 mm diameter at a depth of 200 µm below the corneal surface, followed by a final 90° arc-shaped access cut, oriented at 45° to the lamellar planes and extending to the epithelial surface. Details of the femtosecond laser protocols are given elsewhere^65^. Following the laser procedure, we used a blunt hockey blade tool to separate the access cut and lamellar planes, and surgical forceps to extract the button of native stromal tissue through the access cut. This resulted in a cornea approximately two-thirds the thickness of the normal porcine cornea, mimicking the cornea in advanced keratoconus, albeit with a uniform reduction in thickness across the central 7 mm of cornea.

We thereafter conducted minimally invasive surgery to treat advanced keratoconus. A sterile, packaged BPCDX implant of 280 µm thickness and 10 mm diameter was opened in the operating room, and we cut the device to a 7 mm diameter using a Barron corneal punch trephine. Next, we used surgical forceps to grip the BPCDX, which was then inserted into the intrastromal pocket through the access cut^65^. Although the FLISK surgery was previously performed without the use of surgical sutures^29^, as a precautionary measure we decided to close the region of the access cut in the stroma anterior to the implant using two 10-0 nylon surgical sutures (Supplementary Fig. 2).

Immediately post-operatively, minipigs were placed on a ventilator and once spontaneous breathing resumed, 0.05 mg kg^−1^ intravenous buprenorphine (Temgesic) was given as an analgesic. Post-operatively, topical anesthetic eye drops were again instilled, followed by a combination topical corticosteroid-antibiotic (0.1% dexamethasone + 0.3% tobramycin, Tobrasone eye drops) given three times daily the first post-operative week and twice daily during the following three weeks. Post-operative analgesia consisted of intramuscular injection of 0.05 mg kg^−1^ buprenorphine every 12 h for the first five post-operative days.

### Post-operative assessment and corneal imaging

Six months after surgeries, minipigs were placed under general anesthesia as described above, and we performed in vivo examinations and photo documentation in operated eyes. Examinations consisted of digital photography (Nikon D90 digital camera), anterior segment optical coherence tomography (iVue, Optovue) and in vivo confocal microscopy (Heidelberg Retinal Tomograph 3 with Rostock Corneal Module, Heidelberg Engineering) using a previously described microscopy protocol^66^. Following examinations and data collection, minipigs were euthanized while under general anesthesia and deep sedation, by intramuscular injection of 7 mg kg^−1^ Zoletil and intravenous injection of 100 mg ml^−1^ pentobarbital.

### Histology and Immunohistochemistry

Following euthanasia, rat tissue and porcine corneas (to the limbal margin) were dissected under an operating microscope, embedded in optimal cutting temperature compound, and snap-frozen in liquid nitrogen. Corneas were stored at −80 °C until further use. For histology, rat and pig tissue were thawed, fixed in 4% paraformaldehyde solution, embedded in paraffin and sectioned to a thickness of 4 µm, followed by staining with hematoxylin and eosin (H&E). For immunohistochemistry, sections 10 µm thick were produced using Leica CM1510 cryostat (Leica AB). The resulting sections were mildly fixed with 2% paraformaldehyde (VWR Life Science) for 10 min, permeabilized by incubation in 0.05% Triton X-100 for 10 min and blocked with 5% BSA for 1 h at room temperature. The samples were then incubated overnight at 4 °C with primary antibodies β-III-tubulin (1:100, ab7751, clone TU-20, lot GR3238448-11, Abcam), α-SMA (1:25, ab7817, clone 1A4, lot GR119216-7, Abcam), type III collagen (1:100, Acris AF5810, clone III-53, lot A130097BH) and CD45 (1:100, ab23, clone UCH-L1, lot GR3189280-2, Abcam) in 2.5% BSA. Control sections were incubated with 2.5% BSA alone without the addition of the primary antibody. Sections were washed in PBS-T and visualized by goat anti-mouse IgG (H + L) Alexa Fluor 488 (1:1,000, A32723, polyclonal, RRID AB_2633275, Thermo Fisher Scientific) or DyLight 488 (1:1,000, 35503, polyclonal, RRID AB_844397, Thermo Fisher Scientific) and DyLight 550 (1:1,000, SA5-10173, polyclonal, RRID AB_2556753, Thermo Fisher Scientific) secondary antibodies. Cell nuclei were counterstained with 4′,6-diamidino-2-phenylindole (DAPI); (1:5,000; Sigma). The slides were mounted with a ProLong Diamond antifade reagent (Invitrogen, Thermo Fisher) and imaged with a laser fluorescence confocal microscope (LSM700, Zeiss).

### Ethics statement

BPCDX was implanted intrastromally in human subjects in local investigator-driven pilot feasibility studies in Iran and India. BPCDX was implanted in cases of advanced keratoconus causing severe visual impairment or corneal blindness. Before subject recruitment, ethical approvals for the studies were obtained in Iran (Institutional Review Board, Farabi Hospital, Tehran University of Medical Sciences, Tehran, Ethics Approval Code IR.TUMS.FARABIH.REC.1395.442) and in India (Institute Ethics Committee, All India Institute of Medical Sciences, New Delhi, ref. no. IEC/NP-47/10.04.2015, RP-23/2015). Studies were conducted following the tenets of the Declaration of Helsinki, and signed informed consent was obtained from all participants before inclusion. LinkoCare Life Sciences AB sponsored the pilot studies, which were funded by LinkoCare Life Sciences AB in kind (both sites), Care Group India (Indian study) and the local investigators in India and Iran. No European Union funding was used for clinical activities outside of the EU. The study was an exploratory, non-randomized, non-blinded and non-controlled pilot case series whose primary goal was to test feasibility of a new surgical method and detect possible adverse reactions in the host or in the BPCDX device using different implanted device thicknesses, analogous to an initial dosing study to determine drug tolerance. The rationale for conducting the pilot study was to obtain first feasibility and safety/tolerability data to determine if it would be ethically acceptable to randomize visually impaired patients (a vulnerable group) to possibly receive the experimental treatment and not a standard donor cornea and surgery when available, as would be required for a future randomized controlled trial. Broad dissemination of pilot safety and feasibility data was not initially foreseen, and local investigators initiated surgeries immediately upon receiving local ethical approvals, without pre-registration of the pilot series in a public registry. Interim analysis of pilot data indicated safety and revealed an unexpected efficacy comparable to outcomes of standard transplantation surgery (DALK or PK) with possible additional clinical benefits from the milder surgery. On the basis of the interim data, approvals for randomized controlled studies were granted in the EU (see below). For these reasons, publication of initial pilot feasibility results including details of the proposed minimally invasive surgery would be in the interest of the medical and scientific community, before initiating a randomized controlled trial. To ensure the reporting of the pilot studies in Iran and India would be consistent with best practices for the conduct of investigational studies of medical devices, the ongoing pilot clinical study was registered in the ClinicalTrials.gov database in December 2020 (Clinicaltrials.gov: NCT04653922). The BPCDX manufacturing process and device test results presented here, along with preclinical data and pilot clinical results at 6–12 months of follow-up, were submitted to Regulatory Authorities in Sweden (Medical Products Agency) and the Czech Republic (State Institute for Drug Control), and approvals were granted for a randomized controlled study protocol according to EU Directive 93/42/EEC on Medical Devices (decision 5.1-2018-44565, Sweden; file no. sukls 21920/2020, Czech Republic) and for the BPCDX device manufacturing, sterilization and packaging methods (file no. sukls 21920/2020, Czech Republic). Additionally, an institutional ethical review committee in Sweden reviewed a randomized clinical trial protocol and approved it for use in Sweden (Linköping Regional Ethics Committee, decision 2017/34-31). Because BPCDX device development and preclinical studies were partially funded by the European Union (Project ARREST BLINDNESS, grant no. 667400), before any reporting of results the clinical studies conducted in Iran and India were subject to further review by an independent contracted biomedical ethics expert (H. Draper, University of Warwick, UK) and by an independent Ethics Review Panel at the European Commission, Division of the Director General—Research and Innovation. The outcome of these reviews was favorable, confirming that the conduct of the clinical studies was consistent with accepted ethical practices within the EU and that the studies were conducted without exploitation of the research subjects or local investigators.

### Pilot feasibility study according to ISO 14155

To investigate the safety and feasibility of BPCDX use in LMICs, we conducted pilot studies in Iran (Farabi Eye Hospital, Tehran University of Medical Sciences, Tehran, Iran) and India (All India Institute of Medical Sciences, Dr. R. P. Centre for Ophthalmic Sciences, New Delhi, India). The aim of these pilot studies was to set broad guidelines for the use of the BPCDX device (such as inclusion and exclusion criteria and parameters for intrastromal surgery) to determine the feasibility to implement the proposed treatment in local patient populations, while allowing surgeons the flexibility to adapt to local clinical procedures, protocols and surgeon experience and preferences. Several parameters therefore varied between sites such as the size of BPCDX and size of intrastromal pocket, choice of post-operative medications and timing and conduct of follow-up examinations using instruments and diagnostic methods available to investigators at the local clinics. The goal of these initial case series was to obtain initial safety, feasibility and efficacy data, which if successful, would support the design and implementation of future prospective, randomized, controlled clinical trials.

Ethical permission was granted for the pilot studies to treat up to 20 subjects with advanced keratoconus at each site (40 subjects total), based on the ability to detect adverse events in 10% of cases. We report results of 24-month follow-up of the first 12 patients treated in Iran and the first 8 patients treated in India (20 patients total). For the results reported here, clinical data collection occurred during February 2017–January 2020 in Iran and during November 2016–March 2020 in India. In addition to safety and feasibility of BPCDX implantation in advanced keratoconus using the minimally invasive FLISK procedure, the study sites collected data to enable assessment of possible efficacy in terms of rehabilitation of corneal curvature, corneal thickness and BCVA. A future definitive trial would be based upon occurrence of adverse events (for example, inflammation and rejection) leading to implant removal in a maximum of 10% of cases, along with evidence of efficacy in the form of at least 60% of operated eyes having sustained decrease of keratometry at 6 months, sustained increase in central corneal thickness at 6 months, and minimum visual acuity improvement of one Snellen line of vision at 6 months.

### Subject recruitment and study endpoints

Potential study subjects at both sites were identified on the basis of clinical history, previous consultation visits and fulfillment of study inclusion/exclusion criteria. Potential candidates were contacted by telephone by the study nurses, then sent the study information and consent form by postal mail. If a subject decided to participate in the study, the consent form was signed by the subject and the local investigator-physician in charge of the study, and the subject was formally included. Study participants were not compensated for their participation in the study, monetarily or otherwise. The primary endpoint for this study was to determine the safety and stability of BPCDX implantation, defined as retention of the device without degradation, loss of transparency, inflammation or vascularization of the implant or host cornea during the first 6 post-operative months. Primary outcome measures included safety (determined by maintenance of corneal transparency and absence of rejection, scarring, inflammation or neovascularization detected during clinical examinations up to 6 months post-operatively) and efficacy measures to assess reduction in maximum keratometric power, increase in corneal thickness and improvement in uncorrected and BCVA at 6 months. The secondary endpoint was to determine safety during a 12-month post-operative period, while secondary outcomes were the same safety and efficacy measures as above, but at 12 months. Here we report the longer-term 24-month results for the primary study endpoint in the first 20 operated subjects.

### Inclusion and exclusion criteria for subject selection

Subjects were eligible for study inclusion if the following criteria were all met in at least one eye:

1. Grade 3 or higher advanced keratoconus (according to Amsler–Krumeich classification).
2. No corneal scar.
3. Male or female aged ≥18 years.
4. Subjects indicated for a first corneal transplantation.
5. Corneal thickness (including epithelium) at least 300 µm centrally, as measured by OCT.
6. Signed and dated informed consent.

Patients fulfilling at the selection visit one or more of the following exclusion criteria were not included in the study:

1. Ophthalmic exclusion criteria
  In the affected eye:
  Prior corneal surgery (for example, refractive surgery, cataract, collagen crosslinking, endothelial keratoplasty, etc.).
    In either eye:
  Dry eye/tear film pathology.
  Active ocular infection.
  Glaucoma/ocular hypertension.
  Active corneal ulceration.
  Acute or chronic disease or illness that would increase the operation risk or confound the outcomes of the study (immune-compromised, connective tissue disease, clinically significant atopic disease etc.).
  Any other medical condition that in the judgment of the local clinical investigator was not compatible with the study procedures.
2. Systemic/non-ophthalmic exclusion criteria
  General history judged by the investigator to be incompatible with the study (for example, life-threatening patient condition or other condition where post-operative follow-up may be difficult).
  Known diabetes or other neuro-degenerative disorder (as corneal nerves can be affected leading to impaired wound healing).
3. Exclusion criteria related to general conditions
  Inability of patient to understand the study procedures and thus inability to give informed consent.
  Participation in another clinical study within the last 3 months.
  Already included once in this study (can only be included for one eye).

### Surgical method for clinical case series

We used the minimally invasive FLISK method at both sites. All study subjects were candidates for penetrating or lamellar keratoplasty. The diagnosis of advanced keratoconus was made according to clinical signs (Munson’s sign, Rizutti’s sign) slit-lamp biomicroscopic examination (Vogt’s striae, Fleischer’s ring, apical thinning), corneal topography (skewed asymmetric bow-tie, severe central or inferotemporal steepening, high keratometric power), tomographic signs (abnormal elevation maps, abnormal pachymetry maps, keratoconus detection by Belin/Ambrosio Enhanced Ectasia Display) and refractive results (loss of BCVA). Subjects with refractive errors that could not be corrected with eyeglasses or routine contact lenses, and those who were scleral or mini-scleral contact lens intolerant were included. After the corneal center was marked on the basis of the pupil center, a mid-stromal pocket was created through the marked margins of the temporal cornea using a femtosecond laser. The laser surgical parameters are summarized in Supplementary Fig. 3. Following the laser procedure, the corneal stroma was dissected, taking care to avoid perforation of the Descemet membrane while dissecting the thinnest part of the cornea. Next, BPCDX was removed from the sterile packaging and trephined at 7.5–8.5 mm size and inserted into the intrastromal pocket using surgical forceps to grip the BPCDX across its entire diameter. In Iran, two different thicknesses of BPCDX were used, 280 µm and 440 µm, on the basis of the pre-operative corneal thickness at the thinnest point. Subjects with a thinnest value above 400 μm received 280-μm-thick BPCDX while those with thinnest value below 400 µm received 440-μm-thick BPCDX. In India, all subjects received 280-µm-thick BPCDX regardless of initial corneal thickness. No suturing was performed in any subject. Immediately post-operatively, a soft silicone hydrogel bandage contact lens (Comfilcon A, CooperVision) was placed on the operated cornea for 3 days in all treated subjects.

### Post-operative medications and follow-up examinations

Post-operatively, medications were administered as deemed appropriate by the local surgeons. On the basis of the post-operative wound healing observed in the minipig model (where post-operative medications were administered for 4 weeks) and in a previous rabbit study where the intrastromal implantation model indicated complete wound healing within 8 weeks^29^, an 8-week regimen was instituted. In Iran, patients received artificial tears (carboxymethylcellulose 0.5%) as needed pre-operatively and three times daily post-operatively for 8 weeks, and betamethasone eye drops (0.1%) were given three times daily for 8 weeks post-operatively. Additionally, a bandage contact lens was placed over the operated cornea for 1 week. In India, patients received pre-operative moxifloxacin eye drops (0.5%) three times daily for 3 days prior to surgery. Post-operatively, patients received moxifloxacin eye drops (0.5%) three times daily, prednisolone eye drops (1%) four times daily and artificial tears (carboxymethylcellulose 0.5%) six times daily.

Surgeons assessed the eye immediately following surgery and at 1 day, 1 week and 1, 3, 6 and 12 months post-operatively. An additional 24-month visit was subsequently scheduled. Slit-lamp biomicroscopic evaluation was used to grade the corneal transparency according to a previously published scale^67^, uncorrected visual acuity and BCVA were determined by the Snellen eye chart and spectacles, and expressed in the logMAR scale, Scheimpflug-based anterior segment tomography was performed to assess corneal steepness (Pentacam HR, Oculus, Optikgerate GmbH), and refraction was assessed at various post-operative visits. Corneal thickness was assessed using anterior segment FD-OCT (Iran: Casia, Tomey; India: iVue, OptoVue). Pre-operative and post-operative corneal imaging examinations were performed by the same experienced optometrist at each site. Data was entered into a spreadsheet for later analysis (Microsoft Excel 365 for Windows, 32-bit).

### Statistical analysis

Statistical analysis was performed using Statistical Package for Social Sciences software (v.22, SPSS). Normality in the distribution of the parameters was assessed using the Shapiro–Wilk test. For shelf-life studies, *t*-tests were performed comparing aged to non-aged samples. To examine differences in post-operative values for clinical parameters at 24 months relative to the pre-operative values, a two-tailed paired *t*-test was performed. Where results across two different groups were compared, an independent *t*-test was performed. A two-tailed critical value of *α* < 0.05 was considered statistically significant for all tests, unless otherwise stated. Visual acuity comparisons were made in logMAR units, with each line of improvement in acuity corresponding to a logMAR reduction of 0.1.

### Reporting summary

Further information on research design is available in the Nature Research Reporting Summary linked to this article.

## Online content

Any methods, additional references, Nature Research reporting summaries, source data, extended data, supplementary information, acknowledgements, peer review information; details of author contributions and competing interests; and statements of data and code availability are available at 10.1038/s41587-022-01408-w.

## Supplementary information

Supplementary Information Supplementary Figs. 1–4 and Tables 1–4.

Reporting Summary

## Data Availability

The majority of data analyzed in this study are included in this published article and in the related methods and supplementary information. Source data have been provided for Fig. 1, Extended Data Fig. 1, Table 1, Fig. 2 and Fig. 3. There are no publicly available datasets related to this study. Source data are provided with this paper.

## Source data

Source Data Figure 1 Raw quantitative measurement data.
Source Data Extended Data Fig. 1 Raw quantitative measurement data.
Source Data Table 1 Raw clinical measurement data.
Source Data Figure 2 Raw in vivo minipig image data.
Source Data Figure 3 Raw immunofluorescence tissue section images and raw hematoxylin and eosin stained tissue section images (raw, uncropped versions embedded in file).
