# Supplementary material for: Bioengineered corneal tissue for minimally invasive vision restoration in advanced keratoconus in two clinical cohorts
Source: Nat Biotechnol. 2022 Aug 11;41(1):70–81. doi: 10.1038/s41587-022-01408-w (PMC9849136; doi:10.1038/s41587-022-01408-w)
Supplement: Supplementary file 1 — Supplementary Figs. 1–4 and Tables 1–4. [file 41587_2022_1408_MOESM1_ESM.pdf]

nature portfolio

https://doi.org/10.1038/s41587-022-01408-w

Supplementary information

Bioengineered corneal tissue for minimally invasive vision restoration in advanced keratoconus in two clinical cohorts

In the format provided by the authors and unedited

Supplementary Information for:

Bioengineered corneal tissue for minimally invasive vision restoration in advanced keratoconus in two clinical cohorts

Mehrdad Rafat[1,2,7*], Ph.D., Mahmoud Jabbarvand[3,7], MD, Namrata Sharma[4,7], MD, Maria Xeroudaki[5], MD, Shideh Tabe[1], M.Sc., Raha Omrani[1], M.Sc., Muthukumar Thangavelu[1], Ph.D., Anthony Mukwaya[5], Ph.D., Per Fagerholm[5], MD, Ph.D, Anton Lennikov[5], Ph.D., Farshad Askarizadeh[6], Ph.D., Neil Lagali[5,7*], Ph.D.

[1]LinkoCare Life Sciences AB, Linköping, Sweden

[2] Department of Biomedical Engineering, Linköping University, Linköping, Sweden

[3]Farabi Eye Hospital, Tehran University of Medical Sciences, Tehran, Iran

[4]R.P. Centre for Ophthalmic Sciences, All India Institute of Medical Sciences, New Delhi, India

[5]Division of Ophthalmology, Department of Biomedical and Clinical Sciences, Linköping University, Linköping, Sweden

[6]Department of Optometry, Faculty of Rehabilitation Sciences, Tabriz University of Medical Sciences, Tabriz, Iran.

[7]These authors jointly supervised this work.

*Corresponding authors:

Mehrdad Rafat, Ph.D. (ORCID ID: 0000-0001-6024-4144)
LinkoCare Life Sciences AB
Linköping Science Park, Teknikringen 10, Floor 3,
583 30 Linköping
Sweden
mrafat@linkocare.com
Tel: +46 734613166

Neil Lagali, Ph.D. (ORCID ID: 0000-0003-1079-4361)
Division of Ophthalmology
Department of Biomedical and Clinical Sciences
Linköping University
581 83 Linköping, Sweden
neil.lagali@liu.se
Tel. +46 700850953

This supplementary information consists of:

Supplementary Figure 1

Supplementary Figure 2

Supplementary Figure 3

Supplementary Figure 4

Supplementary Table 1

Supplementary Table 2

Supplementary Table 3

Supplementary Table 4

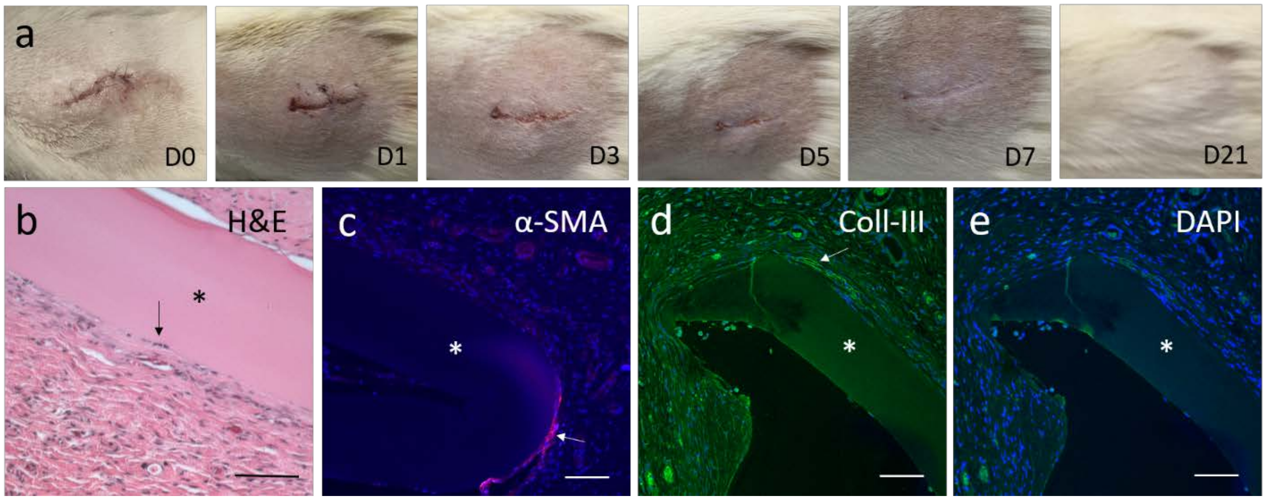

Supplementary Figure 1. Subcutaneous *in vivo* implantation of BPCDX in rats. (**a**) Time series photographs of the surgical site following implantation. (**b**) Histology with hematoxylin and eosin (H&E) staining indicated an intact implant (asterisk indicates BPCDX implant in panels b - e) without signs of material degradation or thinning. Some cells bordered the implant at the interface (arrow), but no strong infiltration of inflammatory cells or new vessel growth at the implantation site was observed. (**c**) α-SMA positive staining was observed at the BPCDX border (arrow), indicating the presence of myofibroblast activity. (**d**) Local deposition of type III collagen (arrow) was present at the BPCDX border, indicating new collagen formation, but without formation of extensive scar tissue. (**e**) DAPI counterstaining indicates presence of cells near and around the implanted region. Images in (**a**) – (**e**) are representative images chosen from 3 independent samples. Scale bar 100µm, all images.

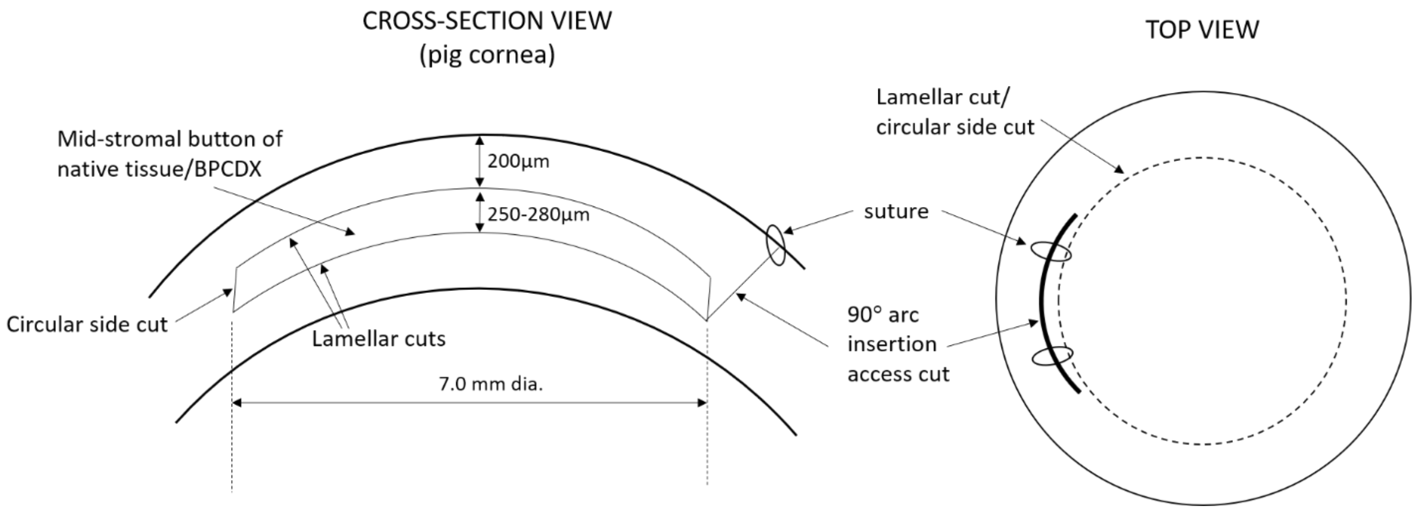

Supplementary Figure 2. Schematic diagram of femtosecond laser-enabled intrastromal keratoplasty (FLISK) surgery for removal of native corneal stromal tissue to mimic keratoconus. A button of the BPCDX slightly thicker (280µm) than the removed button of native tissue (250µm) is implanted intrastromally. The intrastromal removal and insertion of material occurs via a small peripheral access cut at the border of the implant zone. To ensure stability of the cornea, two surgical sutures are placed to seal the access cut region.

a

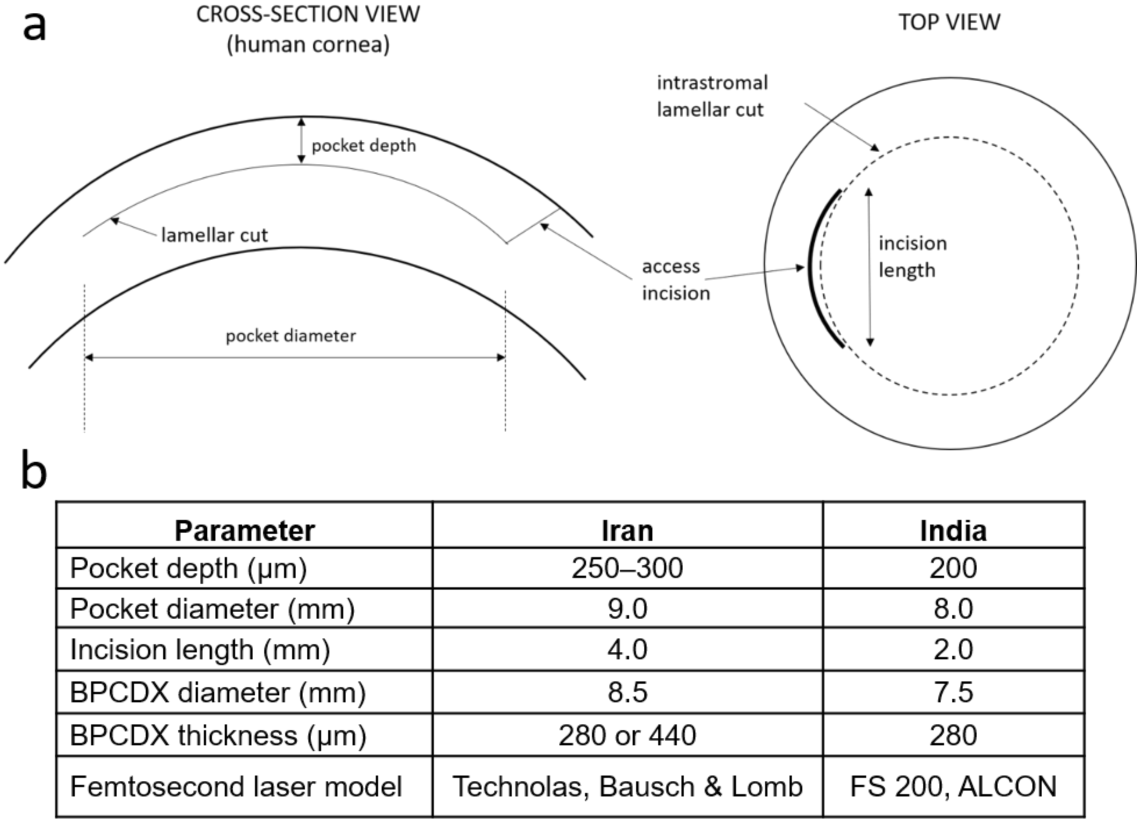

b

Parameter	Iran	India
Pocket depth (μm)	250–300	200
Pocket diameter (mm)	9.0	8.0
Incision length (mm)	4.0	2.0
BPCDX diameter (mm)	8.5	7.5
BPCDX thickness (μm)	280 or 440	280
Femtosecond laser model	Technolas, Bausch & Lomb	FS 200, ALCON

Supplementary Figure 3. Minimally invasive intrastromal surgery parameters for advanced keratoconus. (**a**) Schematic diagram indicating the surgical plan and parameters used for intrastromal surgery in subjects with advanced keratoconus. (**b**) Table of surgical parameter values used at the two study sites.

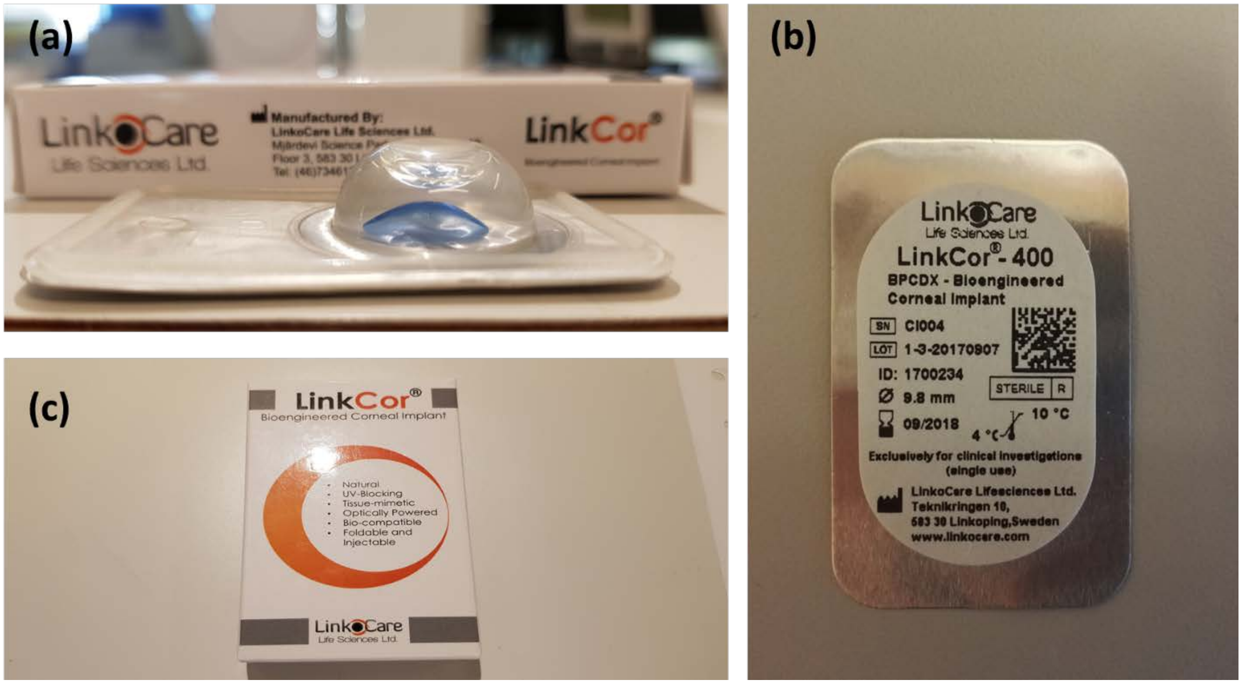

Supplementary Figure 4. Packaging of the sealed and sterilized BPCDX (product name: LinkCor®) for storage or shipping prior to use. (**a**) medical-grade blister pack containing PBS and BPCDX implant (stained blue for visualization purposes only; for medical use, BPCDX is transparent). (b) labels on the back of the blister pack with implant specifications, expiry date, and traceable ID. (c) outer packaging box.

Supplementary Table 1. Comparison of BPCDX material properties with an earlier single-crosslinked collagen-based implant evaluated clinically in keratoconus subjects in a Swedish study.[1-3]

	Swedish study	This study	Footnote
Implant name	RHC-III	BPCDX	1
Chemical crosslinkers	EDC:NHS	EDCM:NHS	2
Crosslinker-to-collagen molar ratio	0.4:1	1:1	3
Initial collagen content by weight (%)	10	18	4
Collagen source	recombinant human; produced in yeast	porcine dermal atelocollagen	5
Collagen used in FDA-approved medical devices	no	yes	6
Collagen type	type III	type I	7
Collagen form	synthetic	natural	8
Atelocollagen composition (%)	< 100	100	9
Manufacturing temperature (°C)	4	Room temperature	10
Photochemical crosslinking method	none	UVA:riboflavin	11
Collagen and crosslinker mixing method	manual	automated	12
Implant sterilization method	1% chloroform in PBS	aseptic manufacturing process; UVA-riboflavin crosslinking; end-stage UVC sterilization	13
Tensile strength (MPa)	0.286 ± 0.062	0.456 ± 0.092	14
Tensile strain (%)	13.9 ± 0.7	12.3 ± 2.6	15
Young's modulus (MPa)	1.749 ± 0.782	6.291 ± 1.640	16
Water content (%)	91.5 ± 0.9	88.8 ± 3.4	17
Optical transmission (%)	95.1 ± 0.05	92.5 ± 1.6 (450 nm)	
		97.7 ± 1.9 (600 nm)	
Refractive index	1.350	1.347	18
Hemolysis test (ISO 10993-4)	not reported	passed	19
Skin sensitization test (ISO 10993-10)	not reported	passed	20
Ocular irritation test (ISO 10993-10)	not reported	passed	21
Sterility test with bacillus pumilus	not reported	SAL < 1 x 10^{-6}	22
Bioburden after sterilization (CFU/ml)	not reported	< 1	23
Endotoxin level (EU/device)	not reported	< 0.091 ± 0.001	24

Footnotes:

1. RHC-III: type III recombinant human collagen; BPCDX: bioengineered porcine construct, double-crosslinked
2. EDC and NHS are zero-length crosslinkers, non-toxic and water-soluble, and not incorporated in the final implant. EDCM (1-Ethyl-3-(3-dimethylaminopropyl)carbodiimide methiodide), used only in

BPCDX, has a higher molecular weight (297.18) relative to EDC (191.70), conferring increased stability and lower reactivity, enabling manufacturing of BPCDX at room temperature, relative to RHC-III which must be manufactured under refrigerated conditions.

3. Higher ratio results in a greater degree of crosslinking and a mechanically stronger implant.
4. Higher collagen content improves implant strength (human cornea is 13.7% collagen).
5. Dermal collagen is more abundant, less expensive and more widely available than recombinant sources.
6. Raw collagen used to manufacture BPCDX is used in approved medical devices.[4,5]
7. The native human cornea is composed predominantly (75%) of type I collagen; type III collagen is associated with inflammation (granulation tissue) and wound healing.[6]
8. Recombinant human collagen requires complex chemical processing and fermentation; porcine dermal collagen is derived from physical tissue processing by dissolution and purification.
9. A portion of the telocollagen sequence is resident on each triple helix of RHC and is reported to be responsible for the antigenicity of collagen.[7,8] In atelocollagen, the immunogenic telocollagen sequence is eliminated.[9]
10. Room temperature production decreases manufacturing time and cost and improves device reproducibility.
11. UVA-riboflavin crosslinking imparts mechanical strength and has antimicrobial and sterilization effects.[10]
12. An automatic pneumatic mixing system is used to ensure homogeneity and reproducibility of BPCDX. A syringe pump system is used to accurately dispense the correct amounts of crosslinkers. For RHC-III, a manual syringe system was used for mixing, and crosslinkers were manually added.[3]
13. Chloroform is difficult to remove, can remain in the RHC-III implant and is cytotoxic.[11] BPCDX is manufactured under aseptic conditions and is exposed to UVA-riboflavin crosslinking as well as an end-stage in-package UVC sterilization procedure.
14. BPCDX has greater tensile strength ($P < 0.001$) than RHC-III implants.
15. No difference in tensile strain ($P = 0.14$).
16. BPCDX has a significantly greater Young's modulus ($P < 0.0001$) relative to RHC-III implants. This improvement brings BPCDX into the range of the Young's modulus of the human cornea, which is 3 - 13 MPa.[12,13]
17. No difference in water content ($P = 0.06$).
18. Hydrated matrix of approximately 90% water has refractive index close to that of water.
19. The hemolytic activity of BPCDX was evaluated by the direct contact method as per ASTM F756-IJ using rabbit blood. The hemolytic index of the test item was comparable to the negative control. The hemolytic index of the test item was between 0 to 2. Hence, the test item was considered as 'non-hemolytic' under the test conditions.
20. BPCDX implants were found to be 'non-sensitizing' after their extracts were intradermally injected into the skin of 10 Guinea pigs and studied over a period of 22 days.
21. Test conducted in six New Zealand white rabbits. A single male rabbit each was used for evaluation of irritation potential of polar and non-polar test item extract. As there was no irritating effect observed up to 24 hours after instillation of the extract, confirmatory test was conducted with two male rabbits each for polar and non-polar test item extract respectively. 0.1 mL of undiluted polar and non-polar test item extract was instilled into lower conjunctival sac of the left eye of each animal. Each animal was examined at 1 (±6 minutes), 24 (±2), 48 (±2) and 72 (±2) hours after instillation. The test item, BPCDX, did not produce any irritant effects to the eyes of New Zealand White rabbits.
22. Performed according to ISO 14937:2016 and ISO 11137-1-3:2017 sterilization standards, as described in Methods. See also Results.
23. See Results.
24. Bacterial endotoxin test performed according to ISO 11979-08, as described in Methods. See also Results.

Supplementary Table 2. Summary of preoperative and 24-month postoperative visual acuity and refractive parameters in both cohorts, on a subject-by-subject basis.

**Iranian Cohort**			BSCVA (logMAR)		Preoperative refraction (Diopters)		24-month refraction (Diopters)	
Subject no.	Age (years)	Sex	Preop	24 months	SER	Astigmatism	SER	Astigmatism
1	18	M	0.20	0.70	2.50	3.50	2.00	6.00
2	39	F	2.00	0.70	-5.75	10.50	-0.50	0.50
3	39	F	1.30	0.15	-13.75	8.00	-3.75	4.50
4	39	F	1.18	0.10	-1.75	6.00	-2.75	3.50
5	36	M	1.30	0.70	-1.00	9.00	-1.00	5.00
6	37	F	1.30	0.40	-1.00	3.00	4.00	8.00
7	18	M	1.00	0.70	1.00	5.00	3.00	6.00
8	18	M	0.70	0.52	-1.00	10.00	4.00	8.00
9	43	M	0.70	0.22	1.50	3.00	5.75	5.50
10	38	M	1.00	0.40	-11.00	10.00	-12.00	0.00
11	18	M	2.00	0.52	-5.00	6.00	-7.63	1.75
12	27	F	2.00	0.40	3.00	8.00	0.75	5.00

**Indian Cohort**				
Subject no.	Age (years)	Sex	Preop	24 months
1	31	F	1.78	0.00
2	26	M	1.48	0.00
3	19	M	1.48	0.17
4	19	M	1.48	0.17
5	31	M	1.78	0.30
6	30	M	1.78	0.17
7	30	M	1.48	0.17
8	27	F	1.78	0.00

BSCVA: best spectacle-corrected visual acuity; BCLVA: best contact-lens-corrected visual acuity; SER: spherical equivalent refraction.

Supplementary Table 3. 24-month visual acuity comparison between the present study cohorts, an earlier Swedish study implanting a biomaterial in keratoconus patients by lamellar keratoplasty,[1,14] and data obtained from the Swedish Corneal Register for two-year follow-up of standard corneal transplantation by penetrating keratoplasty with human donor corneas for keratoconus.

	Current study cohorts			Standard transplantation (donor tissue)[2]
	Iran	India	Sweden[1]	
No. of patients	12	8	10	60
Disease severity, Kmax (D)[3]	69.1 ± 9.0	62.7 ± 7.5	54.5 ± 8.7	Not available
logMAR BSCVA/BCLVA	0.46 ± 0.22[4]	0.12 ± 0.11[5]	0.32 ± 0.26[5]	0.33 ± 0.43[4]
mean Snellen acuity (feet)	20/58	20/26	20/42	20/43
P-value (donor tissue)[6]	0.146	**0.003**		
P-value (Sweden)[7]	0.210	**0.047**		

Footnotes:

[1]Prior Swedish study implanting biomaterial by lamellar keratoplasty, from Refs. 1 and 14
[2]Data from Swedish Corneal Register (SCR), 2-year follow-up of keratoconus subjects after standard corneal transplantation with human donor tissue by penetrating keratoplasty, from Ref. 1. Note that visual acuity parameters are reported in the register, but not keratometry.
[3]Disease severity measured by the maximum preoperative corneal steepness (keratometry) in Diopters. A larger value indicates more advanced disease.
[4]BSCVA (BCLVA not available; not all subjects tolerant to contact lenses after standard corneal transplantation)
[5]BCLVA
[6]P-value relative to standard transplantation, value in bold indicates significant improvement relative to the SCR data
[7]P-value relative to prior Swedish study data, value in bold indicates significant improvement relative to the Swedish study

Supplementary Table 4. Summary of key clinical and preclinical studies evaluating bioengineered corneal tissue as alternatives to human donor tissue for the therapeutic replacement of corneal stroma.

Biomaterial technology	Year published/ reference	Species (indication/model)	Number of subjects (follow-up time, months)	Results (safety and efficacy)
**Clinical Studies**				
Recombinant human collagen type III, single-crosslinked by EDC/NHS*	2010[1], 2014[2]	Human (keratoconus)	10 (24, 48)	Early melting of biomaterial in several patients, corneal thinning in all patients (mean loss of 200µm), stability after initial thinning. Mean BCVA gain of 5.6 lines at 4 years. 3 subjects re-transplanted after 4 years owing to poor refraction/vision.
Recombinant human collagen type III, crosslinked by EDC/NHS and 2-methacryloyloxyethyl phosphorylcholine (synthetic phospholipid)*	2015[15]	Human (acid burns, failed PK)	3 (9 - 12)	Corneal thinning in postoperative period (loss of 20-90µm), patients severely visually impaired at 12 months, persistent loss of transparency and neovascularization.
Decellularized porcine corneal stroma	2015[16]	Human (fungal keratitis)	47 (6)	Reduced corneal transparency in 46 of 47 eyes, persistent corneal neovascularization in 8 eyes. Improvement of > 2 lines of BCVA in 34 eyes.
Recombinant human collagen type III, crosslinked by EDC/NHS and 2-methacryloyloxyethyl phosphorylcholine*	2018[17]	Human (infections, burns, high risk grafts)	6 (14 – 35)	Corneal neovascularization in 4 of 6 eyes, severe edema in 1 case, corneal thinning in 2 eyes.
Decellularized porcine corneal stroma	2019[18]	Human (viral keratitis)	13 (7 – 25)	Corneal neovascularization in 11 of 13 eyes, disintegration of implanted material in 3 eyes, most eyes had some degree of opacity.
Nanostructured fibrin–agarose construct containing human allogeneic stromal keratocytes and cornea epithelial cells	2019[19]	Human (severe corneal ulcers)	16 (24)	No published results available (ClinicalTrials.gov Identifier: NCT01765244). **Requires human donor corneas for stromal and epithelial cell harvesting. Requires human blood donors for fibrin matrix. Shelf life of 6 hours.**
Porcine skin collagen type I, single-crosslinked by EDCM/NHS*†	2020[20]	Human (scarring, leukoma, dystrophies)	6 (12)	Corneal thickness increased in all subjects (20 – 110µm). 1 – 4 lines of BCVA gain in all subjects. No recurrence of dystrophy or scarring. Stromal melting in one subject after 12 months.
Autologous adipose-derived stem cell injection, with and without decellularized human donor stroma	2021[21]	Human (keratoconus)	4-5 (36)	Flattening of keratometry by 2-3 Diopters, corneal thickness increase of 20-30µm, 1-2 lines of gain in BCVA. Liposuction completed in subjects without infection. **Requires human donor corneas and autologous adipose stem cells obtained by liposuction surgery.**

**Preclinical Studies**				
Porcine type I collagen + chitosan, single-crosslinked by EDC/NHS*	2008[22]	Yucatan pigs (healthy cornea)	8 (12)	Variable results: transparency maintained, some neovascularization. Endogenous nerves and cells preserved.
Porcine type I collagen, single-crosslinked by EDC/NHS*†	2015[23]	New Zealand white rabbits (keratoconus model)	10 (2)	Corneal cellular layers and nerves preserved. Corneal thickness maintained or increased. Corneal transparency maintained with mild haze, no neovascularization. Evidence of cellular integration and stromal regeneration.
Fish scale-derived decellularized collagen matrix	2016[24]	New Zealand white rabbits (healthy cornea)	2 (12)	No inflammation or thinning, implant retained. Some scarring noted, no cellular integration with implant. Macrophage infiltration at implant edges.
GelCORE – gelatin methacryloyl (light-activated adhesive)	2019[25]	New Zealand white rabbits (stromal defect model)	3 (0.5)	Rapid degradation of construct *in vitro* in collagenase solution. Histological transformation of implanted material *in vivo* after 2 weeks.
Methacrylated gelatin (GelMA)	2019[26]	New Zealand white rabbits (healthy cornea)	2 (2)	Corneal neovascularization, minimal foreign body reaction, implant degradation *in vivo*.
Bovine type I collagen vitrigel, beta-cyclodextrin modulated	2020[27]	New Zealand white rabbits (healthy cornea)	5 (1)	Full re-epithelialization. Variable results: transparency maintained, scarring, thinning.
Collagen-like peptide + polyethylene glycol + commercial fibrinogen glue*	2020[28]	Göttingen minipig (healthy corneas)	4 (12)	Haze, thinning and neovascularization in implanted corneas. Repeated surgical removal and re-application necessary in all cases. Failure of material attachment in 1 of 4 cases.
Porcine skin collagen type I, single-crosslinked by EDCM/NHS*†	2020[29]	New Zealand white rabbits (keratoconus model)	5 (6)	Intra-stromal implants retained; corneal thickness maintained. Mild haze and mild neovascularization. Macrophage infiltration at implant edges.
Collagen-like peptide + polyethylene glycol + polymeric 2-methacryloyloxyethyl phosphorylcholine*	2021[30]	Göttingen minipig (alkali burn model)	8 (12)	Mild stromal haze in operated eyes at 12 months. Neovascularization in 5 of 8 operated eyes at 12 months. Anterior lamellar keratoplasty with suturing required. Corneal thickness maintained at 12 months.

EDC: 1-[3-(Dimethylamino) propyl]-3-ethylcarbodiimide; EDCM: 1-[3-(Dimethylamino) propyl]-3-ethylcarbodiimide methiodide; NHS:
N-hydroxysuccinimide
*indicates a study co-authored by at least one author of the present study
†indicates a forerunner to the BPCDX used in the present study

References

1. Fagerholm, P. et al. A biosynthetic alternative to human donor tissue for inducing corneal regeneration: 24-month follow-up of a phase 1 clinical study. *Science translational medicine* **2**, 46ra61-46ra61 (2010).
2. Fagerholm, P. et al. Stable corneal regeneration four years after implantation of a cell-free recombinant human collagen scaffold. *Biomaterials* **35**, 2420-2427 (2014).
3. Merrett, K. et al. Tissue-engineered recombinant human collagen-based corneal substitutes for implantation: performance of type I versus type III collagen. *Invest Ophthalmol Vis Sci* **49**, 3887-3894 (2008).
4. Stephens, J.D. & Sarkisian Jr, S.R. The use of collagen matrix (Ologen) as a patch graft in glaucoma tube shunt surgery, a retrospective chart review. *F1000Research* **5** (2016).
5. Snyder, D., Sullivan, N., Margolis, D. & Schoelles, K. Skin substitutes for treating chronic wounds. (2020).
6. Michelacci, Y.M. Collagens and proteoglycans of the corneal extracellular matrix. *Braz. J. Med. Biol. Res.* **36**, 1037-1046 (2003).
7. Miyata, T., Taira, T. & Noishiki, Y. Collagen engineering for biomaterial use. *Clin. Mater.* **9**, 139-148 (1992).
8. Sano, A., Hojo, T., Maeda, M. & Fujioka, K. Protein release from collagen matrices. *Adv Drug Deliv Rev* **31**, 247-266 (1998).
9. Hanai, K., Kojima, T., Ota, M., Onodera, J. & Sawada, N. Effects of atelocollagen formulation containing oligonucleotide on endothelial permeability. *J Drug Deliv* **2012**, 245835 (2012).
10. Tabibian, D., Mazzotta, C. & Hafezi, F. PACK-CXL: Corneal cross-linking in infectious keratitis. *Eye and Vision* **3**, 1-5 (2016).
11. Simpson, F. et al. AB085. E-beam sterilization of recombinant human collagen-phosphorylcholine corneal implants for transplantation. *Annals of Eye Science* **3**, AB085 (2018).
12. Jue, B. & Maurice, D.M. The mechanical properties of the rabbit and human cornea. *J Biomech* **19**, 847-853 (1986).
13. Crabb, R.A., Chau, E.P., Evans, M.C., Barocas, V.H. & Hubel, A. Biomechanical and microstructural characteristics of a collagen film-based corneal stroma equivalent. *Tissue Eng* **12**, 1565-1575 (2006).
14. Ong, J.A. et al. 3D Corneal Shape After Implantation of a Biosynthetic Corneal Stromal Substitute. *Invest Ophthalmol Vis Sci* **57**, 2355-2365 (2016).
15. Buznyk, O. et al. Bioengineered corneas grafted as alternatives to human donor corneas in three high-risk patients. *Clinical and translational science* **8**, 558-562 (2015).
16. Zhang, M.C. et al. Lamellar keratoplasty treatment of fungal corneal ulcers with acellular porcine corneal stroma. *American Journal of Transplantation* **15**, 1068-1075 (2015).
17. Islam, M.M. et al. Biomaterials-enabled cornea regeneration in patients at high risk for rejection of donor tissue transplantation. *NPJ Regenerative medicine* **3**, 1-10 (2018).
18. Zheng, J. et al. Short-term results of acellular porcine corneal stroma keratoplasty for herpes simplex keratitis. *Xenotransplantation* **26**, e12509 (2019).
19. Rico-Sánchez, L. et al. Successful development and clinical translation of a novel anterior lamellar artificial cornea. *Journal of Tissue Engineering and Regenerative Medicine* **13**, 2142-2154 (2019).
20. Khodaparast, M. et al. Sutureless Femtosecond Laser-Assisted Anterior Lamellar Keratoplasty Using a Bioengineered Cornea as a Viable Alternative to Human Donor Transplantation for Superficial Corneal Opacities. *Cornea* **39**, 1184-1189 (2020).
21. El Zarif, M. et al. Corneal Stromal Regeneration Therapy for Advanced Keratoconus: Long-term Outcomes at 3 Years. *Cornea* **40**, 741-754 (2021).
22. Rafat, M. et al. PEG-stabilized carbodiimide crosslinked collagen-chitosan hydrogels for corneal tissue engineering. *Biomaterials* **29**, 3960-3972 (2008).
23. Koulikovska, M. et al. Enhanced regeneration of corneal tissue via a bioengineered collagen construct implanted by a nondisruptive surgical technique. *Tissue Eng Part A* **21**, 1116-1130 (2015).

24. Van Essen, T. et al. Biocompatibility of a fish scale-derived artificial cornea: cytotoxicity, cellular adhesion and phenotype, and in vivo immunogenicity. *Biomaterials* **81**, 36-45 (2016).
25. Sani, E.S. et al. Sutureless repair of corneal injuries using naturally derived bioadhesive hydrogels. *Science advances* **5**, eaav1281 (2019).
26. Kilic Bektas, C. et al. Methacrylated gelatin hydrogels as corneal stroma substitutes: in vivo study. *J Biomater Sci Polym Ed* **30**, 1803-1821 (2019).
27. Wang, X. et al. Multifunctional synthetic Bowman's membrane-stromal biomimetic for corneal reconstruction. *Biomaterials* **241**, 119880 (2020).
28. McTiernan, C.D. et al. LiQD Cornea: Pro-regeneration collagen mimetics as patches and alternatives to corneal transplantation. *Science advances* **6**, eaba2187 (2020).
29. Xeroudaki, M. et al. A porous collagen-based hydrogel and implantation method for corneal stromal regeneration and sustained local drug delivery. *Scientific Reports* **10**, 1-18 (2020).
30. Simpson, F.C. et al. Collagen analogs with phosphorylcholine are inflammation-suppressing scaffolds for corneal regeneration from alkali burns in mini-pigs. *Communications biology* **4**, 1-15 (2021).
