# Supplementary material for: Bioengineered corneal tissue for minimally invasive vision restoration in advanced keratoconus in two clinical cohorts
Source: Nat Biotechnol. 2022 Aug 11;41(1):70–81. doi: 10.1038/s41587-022-01408-w (PMC9849136; doi:10.1038/s41587-022-01408-w)
Supplement: Supplementary file 2 — Reporting Summary [file 41587_2022_1408_MOESM2_ESM.pdf]

nature research

Corresponding author(s): Mehrdad Rafat and Neil Lagali

Last updated by author(s): Jun 2, 2022

Reporting Summary

Nature Research wishes to improve the reproducibility of the work that we publish. This form provides structure for consistency and transparency in reporting. For further information on Nature Research policies, see our Editorial Policies and the Editorial Policy Checklist.

Statistics

For all statistical analyses, confirm that the following items are present in the figure legend, table legend, main text, or Methods section.

n/a	Confirmed	
☐	☒	The exact sample size (*n*) for each experimental group/condition, given as a discrete number and unit of measurement
☐	☒	A statement on whether measurements were taken from distinct samples or whether the same sample was measured repeatedly
☐	☒	The statistical test(s) used AND whether they are one- or two-sided<br>*Only common tests should be described solely by name; describe more complex techniques in the Methods section.*
☒	☐	A description of all covariates tested
☒	☐	A description of any assumptions or corrections, such as tests of normality and adjustment for multiple comparisons
☐	☒	A full description of the statistical parameters including central tendency (e.g. means) or other basic estimates (e.g. regression coefficient) AND variation (e.g. standard deviation) or associated estimates of uncertainty (e.g. confidence intervals)
☐	☒	For null hypothesis testing, the test statistic (e.g. F, t, r) with confidence intervals, effect sizes, degrees of freedom and P value noted<br>*Give P values as exact values whenever suitable.*
☒	☐	For Bayesian analysis, information on the choice of priors and Markov chain Monte Carlo settings
☒	☐	For hierarchical and complex designs, identification of the appropriate level for tests and full reporting of outcomes
☒	☐	Estimates of effect sizes (e.g. Cohen's *d*, Pearson's *r*), indicating how they were calculated

Our web collection on statistics for biologists contains articles on many of the points above.

Software and code

Policy information about availability of computer code

Data collection	Data was collected using commercial software for recording results, i.e., Windows-based Microsoft Excel Spreadsheets (Excel for Microsoft 365, 32-bit version. Mechanical test data was collected via the Instron 5943 testing system's BlueHill Version 3 software.
Data analysis	Statistical analysis was performed using the commercially available Statistical Package for Social Sciences software version 22 (SPSS Inc., Chicago, IL, USA).

For manuscripts utilizing custom algorithms or software that are central to the research but not yet described in published literature, software must be made available to editors and reviewers. We strongly encourage code deposition in a community repository (e.g. GitHub). See the Nature Research guidelines for submitting code & software for further information.

Data

Policy information about availability of data

All manuscripts must include a data availability statement. This statement should provide the following information, where applicable:

- Accession codes, unique identifiers, or web links for publicly available datasets
- A list of figures that have associated raw data
- A description of any restrictions on data availability

The majority of data analyzed in this study are included in this published article and in the related methods and supplementary information. Source data have been provided for Figure 1, Extended Data Figure 1, Table 1, Figure 2 and Figure 3. There are no publicly available data sets related to this study.

Field-specific reporting

Please select the one below that is the best fit for your research. If you are not sure, read the appropriate sections before making your selection.

☒ Life sciences ☐ Behavioural & social sciences ☐ Ecological, evolutionary & environmental sciences

For a reference copy of the document with all sections, see nature.com/documents/nr-reporting-summary-flat.pdf

Life sciences study design

All studies must disclose on these points even when the disclosure is negative.

Sample size

Sample size calculations were not used as the primary goal was to test feasibility and safety in this first-in-man study (this was a non-comparative study). For the minipig study, 5 control and 5 BPCDX-implanted animals were deemed sufficient to test feasibility and safety of the procedure and stability of the BPCDX relative to native stromal tissue, based on our prior preclinical studies implanting similar materials (Refs 29-31, 40, 45) and based on preclinical studies published by other research teams (see Supplementary Table 4). Based on lack of adverse events in prior rabbit studies using a similar implantation technique, it was reasoned that 10 minipigs would be sufficient to determine feasibility in a large-animal model of corneal intrastromal surgery. Apart from biocompatibility, maintenance of corneal thickness and transparency and absence of major adverse events (corneal thinning, melting, loss of transparency, vascularization, etc.) that could be detected using the chosen sample size, a larger sample size was not deemed useful as the preclinical models are limited in their ability to predict surgical outcome and postoperative complications in humans, owing to anatomic differences and differences in postoperative administration of medications in the eye (compliance). The main goals of the minipig study were to test feasibility of implementation using standard surgical equipment and protocols, and to detect possible adverse events during surgery (eg., perforation of the cornea) and postoperatively (melting of device or cornea, scarring, neovascularization). The outcomes were primarily qualitative. For the pilot study, 20 subjects per site was deemed sufficient to determine feasibility of BPCDX intrastromal implantation in a sufficiently broad group of subjects with keratoconus, to determine if adverse events (i.e., device melting, corneal thinning, immune rejection) could occur frequently (more than 10% of the time, i.e., in more than one subject per site). This represents a study size larger than most prior clinical studies in the field (see Supplementary Table 4).

Data exclusions

Data from the pilot case series are final data after 24 months of follow-up in a total of 20 subjects (first 20 subjects operated). All data were available at the time of manuscript preparation, with the exception of some of the clinical parameters at 24 month follow-up for subject 1 from the Iranian cohort (missing data, see Source Data File for Table 1).

Replication

Reproducibility of BPCDX properties and test results was confirmed by testing multiple devices and batches as part of the manufacturing quality management system and as part of certification testing. Replication was done on independent batches at least 3 times. All replicated tests indicated the device passed threshold success criteria. Biomaterial biological effects were tested in multiple in vitro and animal models by third-party certification services. The certification testing was done using multiple independent samples per test, typically 3 - 5. All certification testing was successful (devices passed predetermined thresholds). Preclinical results were based on 5 biological replicates per treatment group. The clinical pilot study was replicated at two sites, in Iran and India. All replications successfully verified reproducibility of the results, to the level of the standard deviations reported.

Randomization

For materials and certification testing, samples were subjected to testing/analysis in random order (i.e., randomly selected from a batch and not based on a priori knowledge of measured properties). For the rat subcutaenous implantations, randomly selected materials were implanted into rats chosen at random from a given litter. For the minipig study, animals were assigned randomly to treatment groups, and surgeries (BPCDX or native tissue implantation) were performed in random order. Human subjects in both pilot case series deemed consistent with inclusion/exclusion criteria were included and no control group was used for clinical pilot studies thus randomization was not applicable.

Blinding

Blinding was not relevant for materials and certification testing as no group comparisons were made during testing (test criteria were absolute). No blinding was used in the rat subcutaneous implantations because no control comparisons were used and results were qualitative. No blinding was used in the minipig study because this was not possible; the identity of the implanted device (versus native tissue) was clearly discernible to the surgeon by visual inspection, and was clearly discernible in postoperative examinations by OCT and confocal microscopic imaging. The clinical pilot study was open-label without a control group, thus blinding was not applicable during data collection. Analysis of data was completed separately for each study site where a single intervention was performed, thus blinding during data analysis was not applicable.

Reporting for specific materials, systems and methods

We require information from authors about some types of materials, experimental systems and methods used in many studies. Here, indicate whether each material, system or method listed is relevant to your study. If you are not sure if a list item applies to your research, read the appropriate section before selecting a response.

Materials & experimental systems

n/a	Involved in the study
☐	☒ Antibodies
☐	☒ Eukaryotic cell lines
☒	☐ Palaeontology and archaeology
☐	☒ Animals and other organisms
☐	☒ Human research participants
☐	☒ Clinical data
☒	☐ Dual use research of concern

Methods

n/a	Involved in the study
☒	☐ ChIP-seq
☒	☐ Flow cytometry
☒	☐ MRI-based neuroimaging

Antibodies

Antibodies used	Primary antibodies β-III tubulin (1:100, ab7751, clone TU-20, lot GR3238448-11, Abcam, Cambridge UK); α-SMA (1:25, ab7817, clone 1A4, lot GR119216-7, Abcam); type III collagen (1:100, Acris AF5810, clone III-53, lot A130097BH, Germany), and CD45 (1:100, ab23, clone UCH-L1, lot GR3189280-2, Abcam). Secondary antibodies were goat anti-mouse IgG (H + L): Alexa Fluor 488 (1:1000, A32723, polyclonal, RRID AB_2633275, Thermo Fisher Scientific, MA, USA) or DyLight 488 (1:1000, 35503, polyclonal, RRID AB_844397, Thermo Fisher Scientific, USA) and DyLight 550 secondary antibodies (1:1000, SA5-10173, polyclonal, RRID AB_2556753, Thermo Fisher Scientific, USA)
Validation	Validation of beta III tubulin antibody: https://www.abcam.com/beta-iii-tubulin-antibody-tu-20-neuronal-marker-ab7751.html<br>Validation of alpha smooth muscle actin antibody: https://www.abcam.com/alpha-smooth-muscle-actin-antibody-1a4-ab7817.html<br>Validation of collagen III antibody: https://m1.acris-antibodies.com/pdf/AF5810.pdf, also validated by us in human tissue samples.<br>Validation of CD45 antibody: https://www.abcam.com/cd45ro-antibody-uch-l1-ab23.html

Eukaryotic cell lines

Policy information about cell lines

Cell line source(s)	HCE-2 (50.B1) human corneal epithelial cell line lot 70015331, sourced from ATCC, Teddington, Middlesex, UK
Authentication	The cell line was authenticated by short term repeat analysis confirming the unique human DNA profile of corneal epithelial cells across 9 human STR loci, cytochrome C oxidase I analysis for species determination, and cell morphology and growth properties (adherent cells with polygonal shape and clear, sharp boundaries between cells).
Mycoplasma contamination	The cell line tested negative for mycoplasma contamination using three methods: Hoechst DNA stain, agar culture and PCR.
Commonly misidentified lines<br>(See ICLAC register)	No commonly misidentified cell lines were used.

Animals and other organisms

Policy information about studies involving animals; ARRIVE guidelines recommended for reporting animal research

Laboratory animals	Rats: male Wistar rats aged 8 weeks at time of surgery. MInipigs: Female Göttingen minipigs aged 6 months at time of surgery.
Wild animals	The study did not involve wild animals.
Field-collected samples	The study did not involve samples collected from the field.
Ethics oversight	Rat experimental protocols were approved by the Linköping Animal Ethical Review Board (Permit ID 585), with procedures in compliance with EU Directive 2010/63/EU on the protection of animals used for scientific purposes.<br>Minipig experimental protocols were approved by the Linköping Animal Ethical Review Board (Permit ID numbers 153 and 37-16) and adhered to the guidelines of the ARVO Statement for the Use of Animals in Ophthalmic and Visual Research and the Directive 2010/63/EU.

Note that full information on the approval of the study protocol must also be provided in the manuscript.

Human research participants

Policy information about studies involving human research participants

Population characteristics	The age and sex of each included participant is given in tabular form in Supplementary Table 2. All patients fulfilled the study inclusion criteria of being aged 18 years or older and having in one 'study eye' advanced keratoconus (Amsler–Krumeich classification Grade 3 or 4), without corneal scar, indicated for a first corneal transplantation, and with corneal thickness (including epithelium) of at least 300μm centrally, as measured by optical coherence tomography.
Recruitment	Potential study subjects at both sites were identified based on clinical history, prior consultation visits and fulfilment of study inclusion/exclusion criteria. A complete list of inclusion and exclusion criteria is given in the manuscript. Potential candidates

were contacted by telephone by the study nurses, and then the study information and consent form were sent by postal mail. If a subject decided to participate in the study, the consent form was signed by the patient and the local investigator-physician in charge of the study, and the subject was formally included. Study participants were not compensated for their participation in the study, monetarily or otherwise. Study participants were included consecutively as fulfilling the study criteria; however, possible bias may have been introduced by the surgeon's assessment of the likelihood of patient willingness to participate and complete follow-up examinations based on subject age, status of the cornea, and other clinical characteristics. Acute need for the surgery and lack of alternative treatment options may also have introduced a bias towards subject inclusion. These potential recruitment biases (not explicitly reported or controlled for in the study) may have resulted in differing outcomes at the different clinical sites and/or a potentially skewed age, sex, or preoperative corneal thickness, steepness or visual acuity distribution. The result of such biases, if present, could limit the generalizability of the results to keratoconus populations in the respective countries.

Ethics oversight

Prior to subject recruitment, ethical approvals for the studies were obtained in Iran (by the Institutional Review Board, Farabi Hospital, Tehran University of Medical Sciences, Tehran, Ethics Approval Code IR.TUMS.FARABIH.REC.1395.442) and in India (by the Institute Ethics Committee, All India Institute of Medical Sciences, New Delhi, Ref. No. IEC/NP-47/10.04.2015, RP-23/2015). A related study protocol was further evaluated and approved by the Linköping Regional Ethics Committee, for use in Sweden (Decision 2017/34-31) and the Swedish Medical Products Agency (Regulatory authority in Sweden, Decision 5.1-2018-44565). Because of the potential for ethical issues arising from research conducted in low-to-middle income countries, further ethical oversight was conducted by an independent contracted Biomedical Ethics expert (Prof. Heather Draper, University of Warwick, UK) and by an ethical oversight committee of the European Commission (Division of the Director General – Research and Innovation).

Note that full information on the approval of the study protocol must also be provided in the manuscript.

Clinical data

Policy information about clinical studies

All manuscripts should comply with the ICMJE guidelines for publication of clinical research and a completed CONSORT checklist must be included with all submissions.

Clinical trial registration

Clinicaltrials.gov: NCT04653922

Study protocol

Study protocol is given in the article and in the clinicaltrials.gov record.

Data collection

Data collection was conducted at both sites in Tehran and New Delhi, at the eye clinics where the surgeries were performed. Recruitment began in 2016 and is ongoing. Data collection occurred preoperatively and at postoperative months 6, 12, and 24 in addition to any data the local investigators deemed necessary to collect outside these time points. For the results reported in the article, clinical data collection occurred during Feb 2017 - Jan 2020 in Iran and during Nov 2016 - Mar 2020 in India.

Outcomes

Primary outcome measures: safety (maintenance of corneal transparency, absence of rejection, scarring, inflammation, or neovascularization as determined by clinical examination at a minimum of 6 months postoperatively), efficacy in reduction of corneal steepness (a sustained decrease in maximum keratometry value at minimum 6 months postoperatively as measured by clinical corneal topography), efficacy in reversal of corneal thinning (a sustained increase in central corneal thickness for at least 6 months postoperatively as measured by clinical optical coherence tomography), efficacy in improvement of visual acuity (an increase in spectacle or contact lens-corrected visual acuity as determined by clinical refraction using a Snellen chart).
Secondary outcomes are the same as the above primary outcomes, however the difference being evaluation at a minimum of 12 months postoperative.
As data at a longer follow-up time of 24 months for the first 20 subjects was available at the time of manuscript revision, on advice from the reviewers and handling editor, we reported the 24 month data from both cohorts.
