# Supplementary material for: Bioengineered corneal tissue for minimally invasive vision restoration in advanced keratoconus in two clinical cohorts
Source: Nat Biotechnol. 2022 Aug 11;41(1):70–81. doi: 10.1038/s41587-022-01408-w (PMC9849136; doi:10.1038/s41587-022-01408-w)
Supplement: Source Data Figure 2 — Raw in vivo minipig image data. [file 41587_2022_1408_MOESM6_ESM.pptx]

## Slide 1
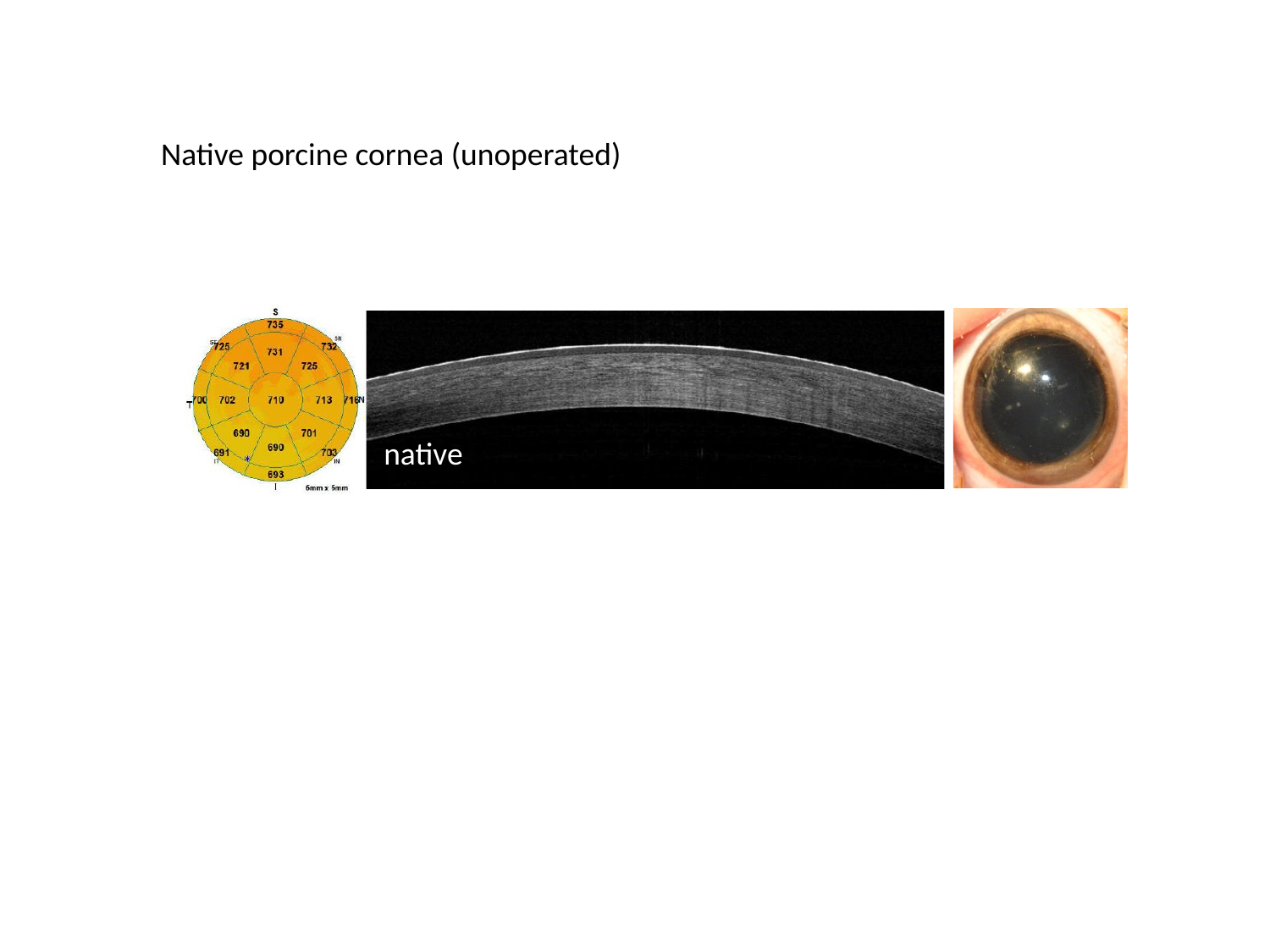

Native porcine cornea (unoperated)
native

## Slide 2
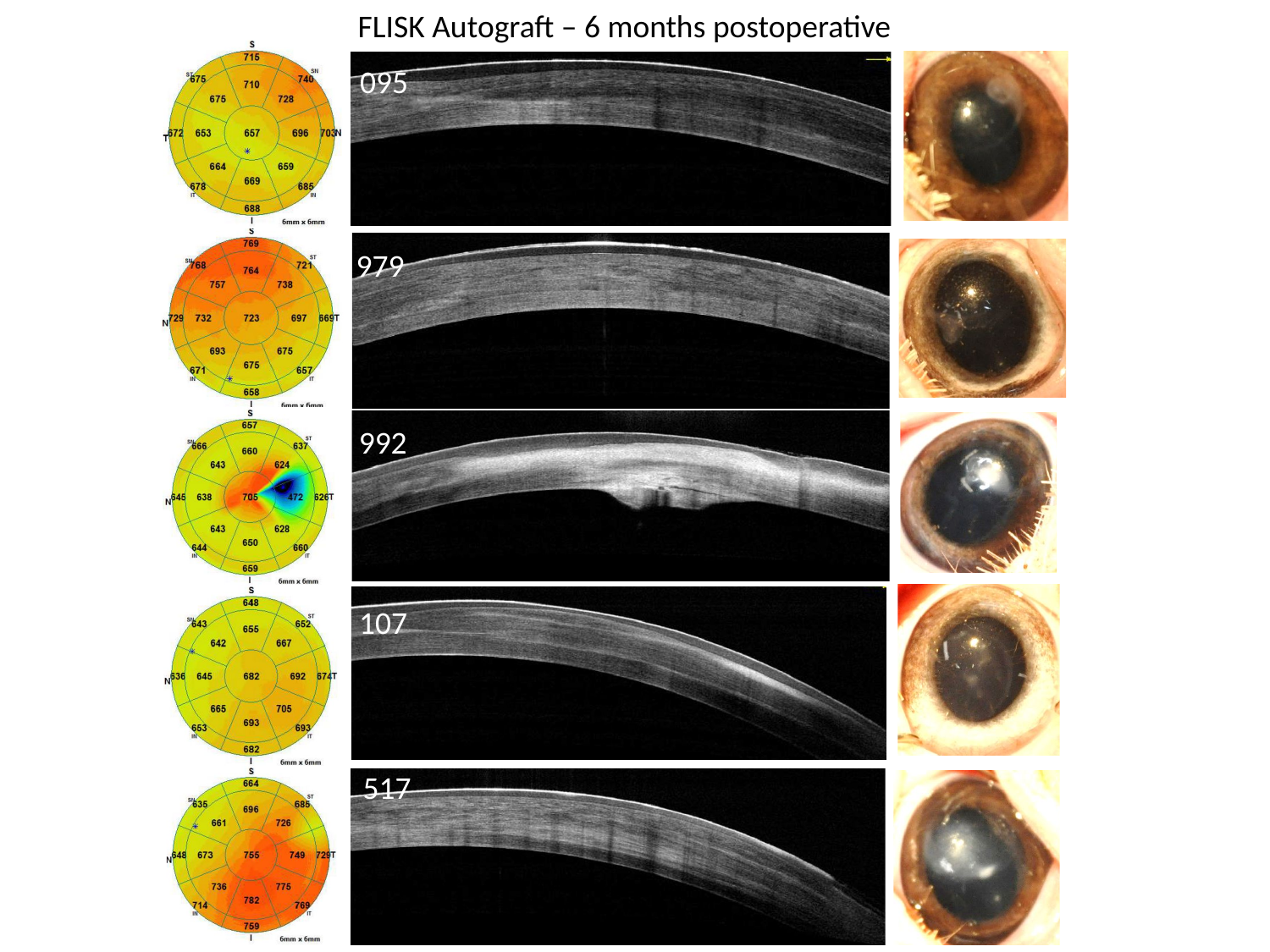

FLISK Autograft – 6 months postoperative
095
979
992
107
517

## Slide 3
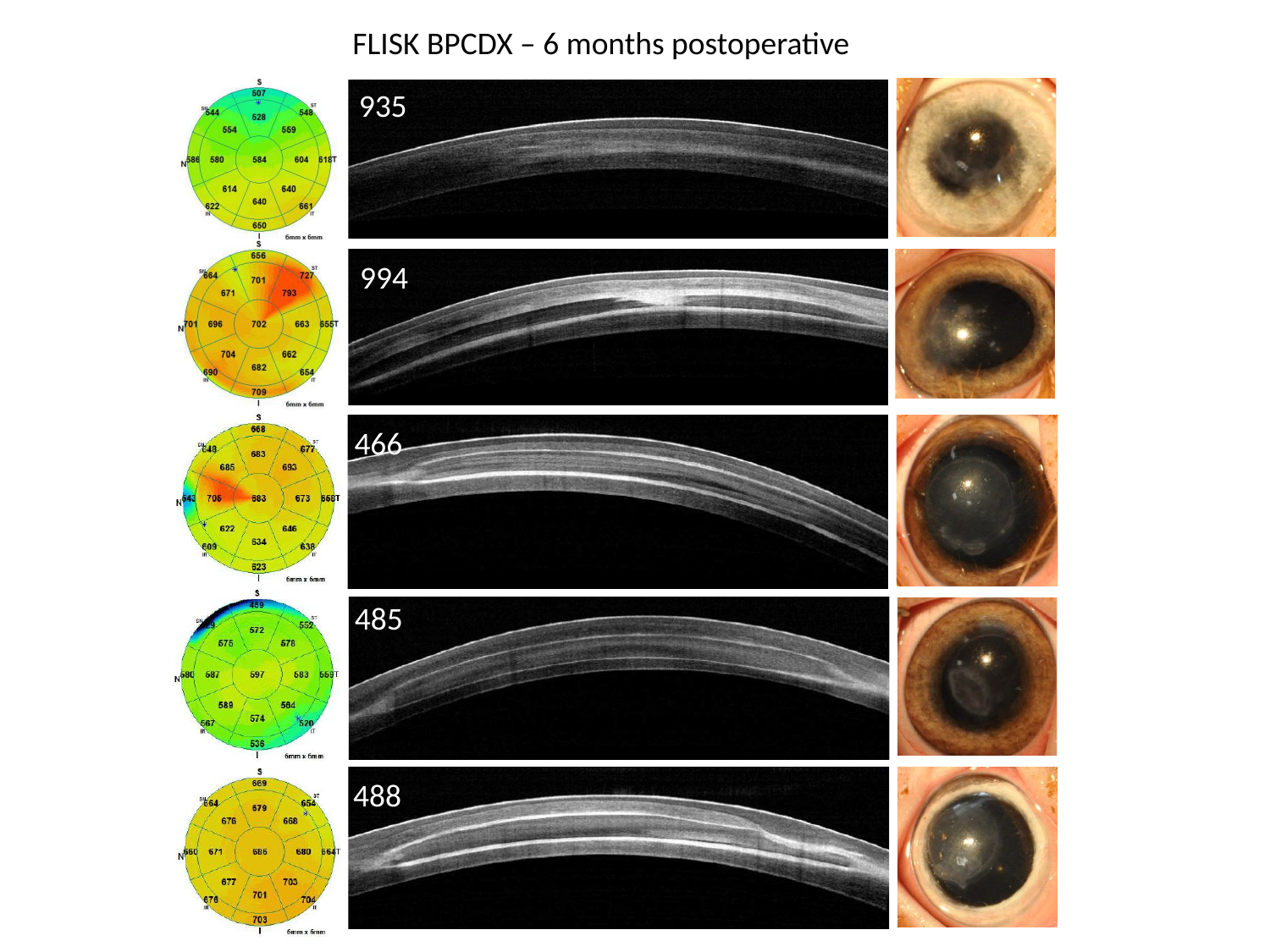

FLISK BPCDX – 6 months postoperative
935
994
466
485
488

## Slide 4
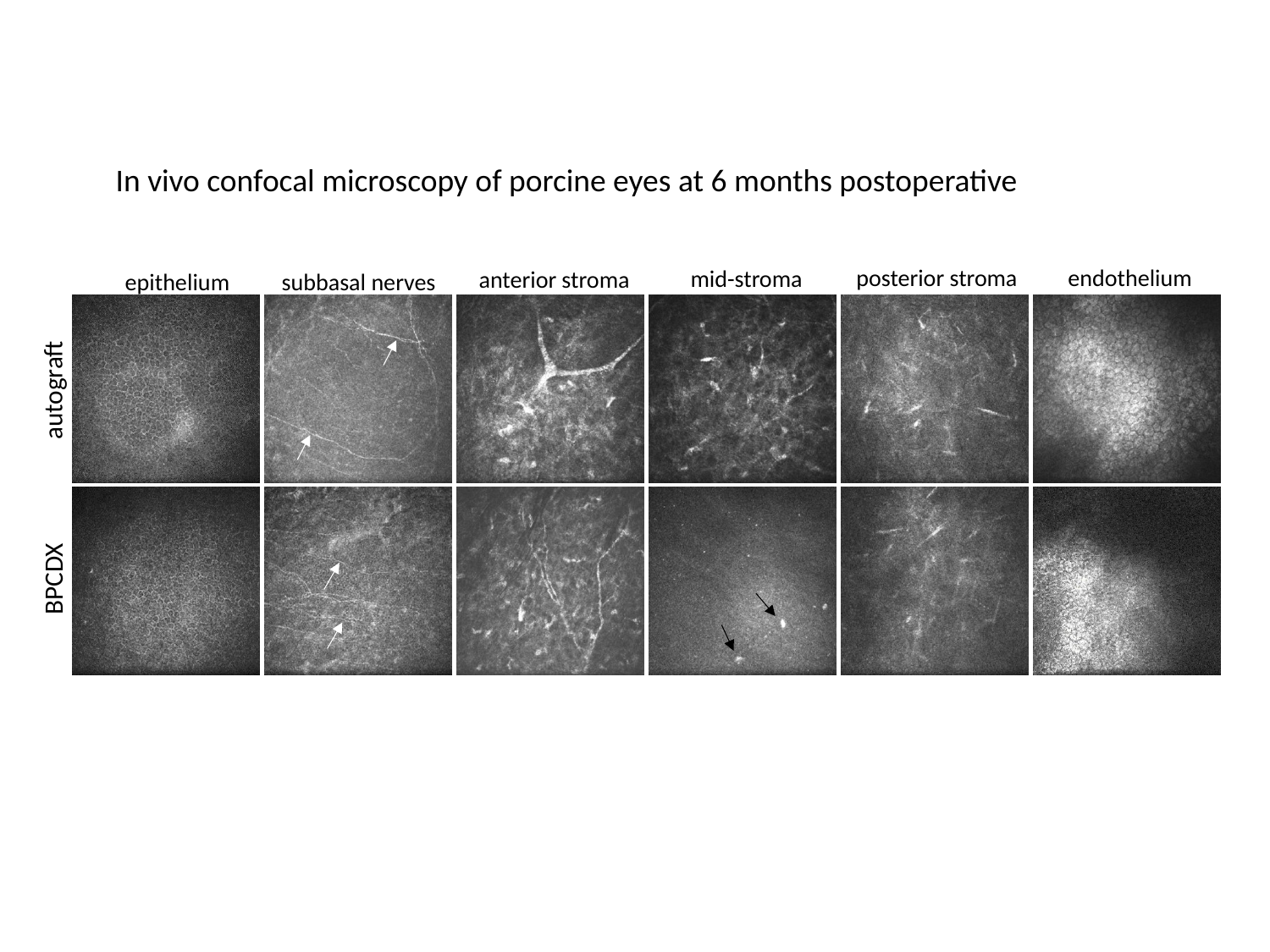

In vivo confocal microscopy of porcine eyes at 6 months postoperative
posterior stroma
endothelium
mid-stroma
anterior stroma
subbasal nerves
epithelium
autograft
BPCDX
