# Supplementary figures and images for: Bioengineered corneal tissue for minimally invasive vision restoration in advanced keratoconus in two clinical cohorts
Source: Nat Biotechnol. 2022 Aug 11;41(1):70–81. doi: 10.1038/s41587-022-01408-w (PMC9849136; doi:10.1038/s41587-022-01408-w)

## Slide 1
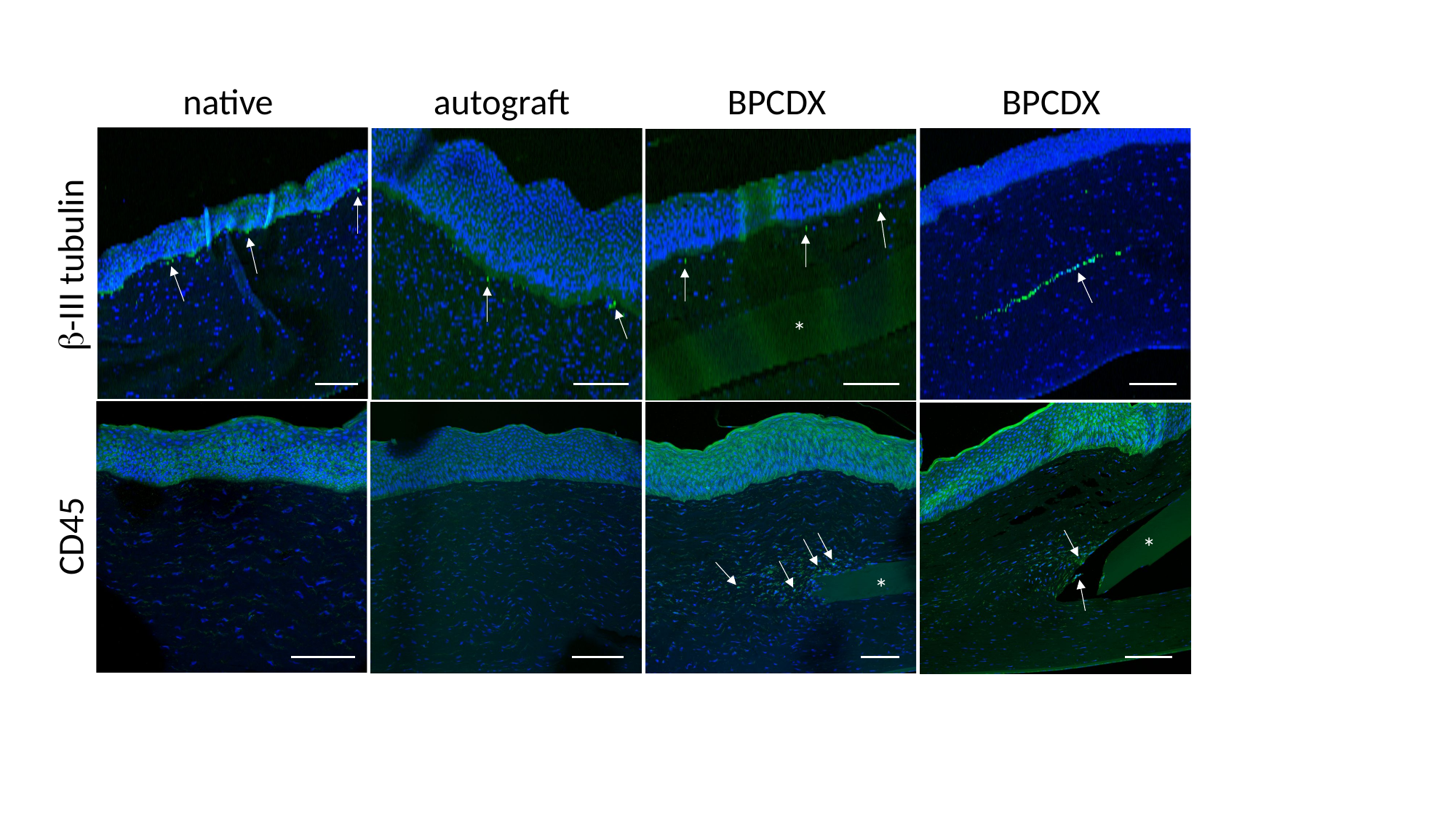

native
autograft
BPCDX
BPCDX
β-III tubulin
*
CD45
*
*

## Slide 2
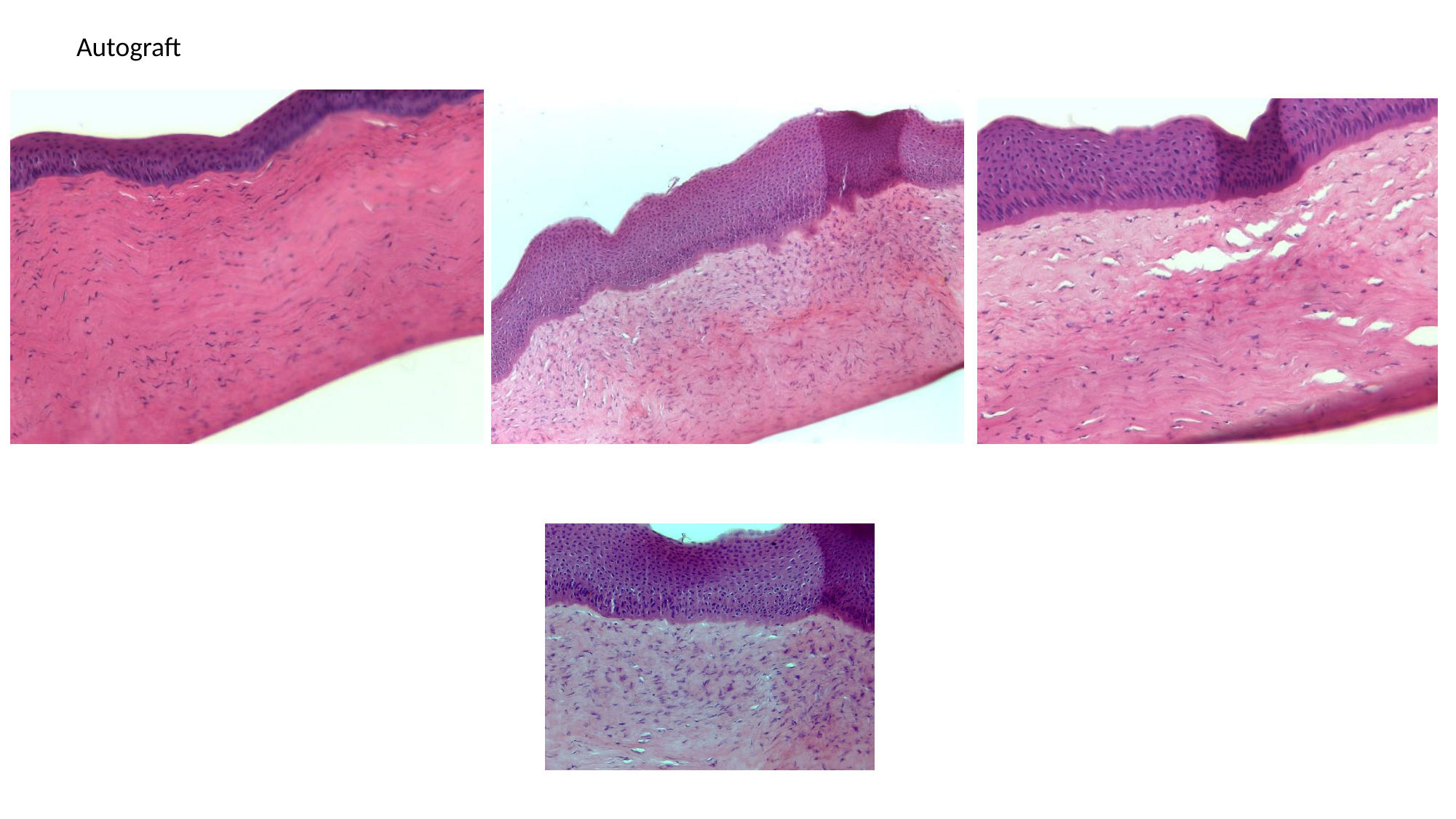

Autograft

## Slide 3
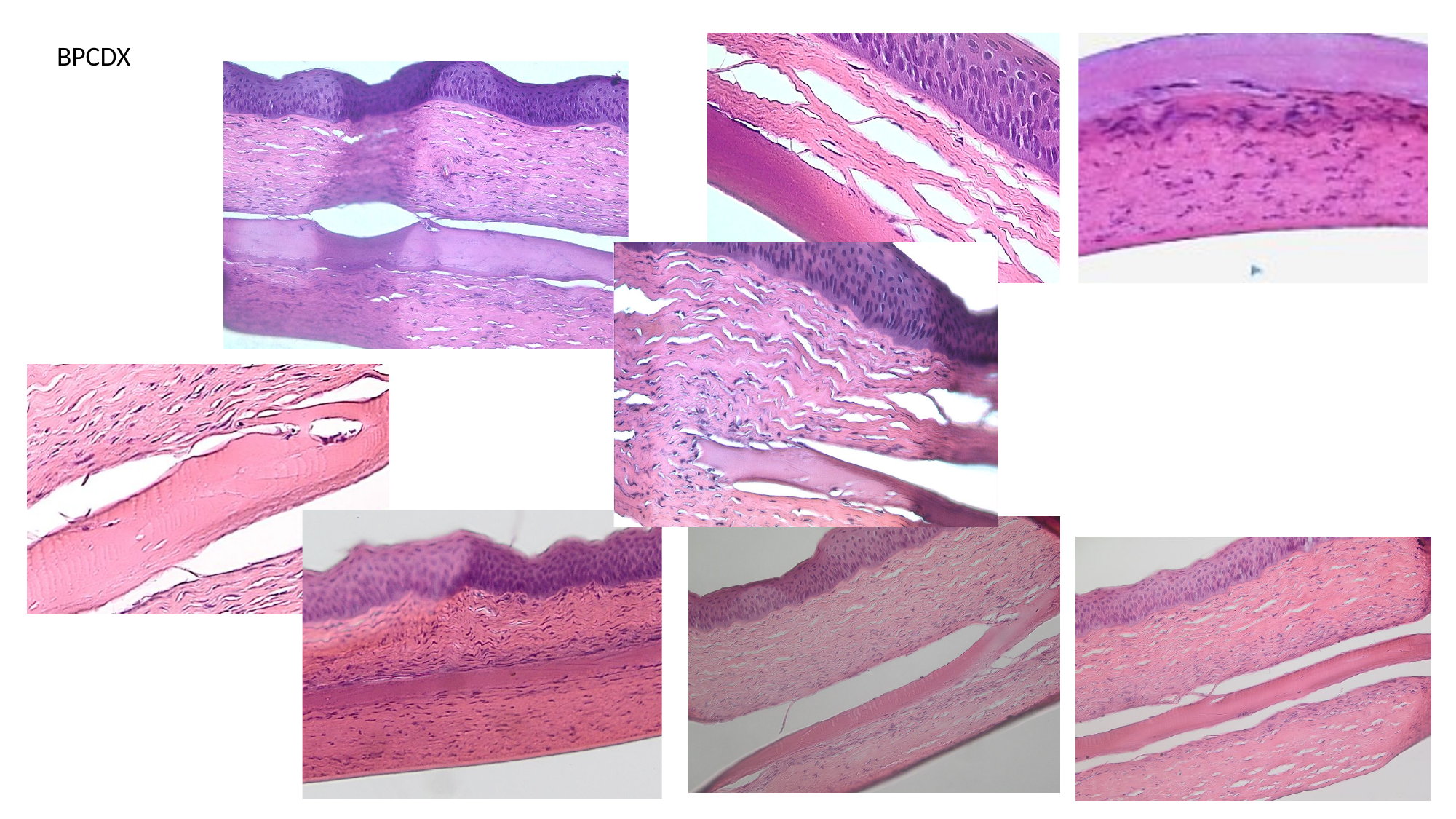

BPCDX

## Slide 4
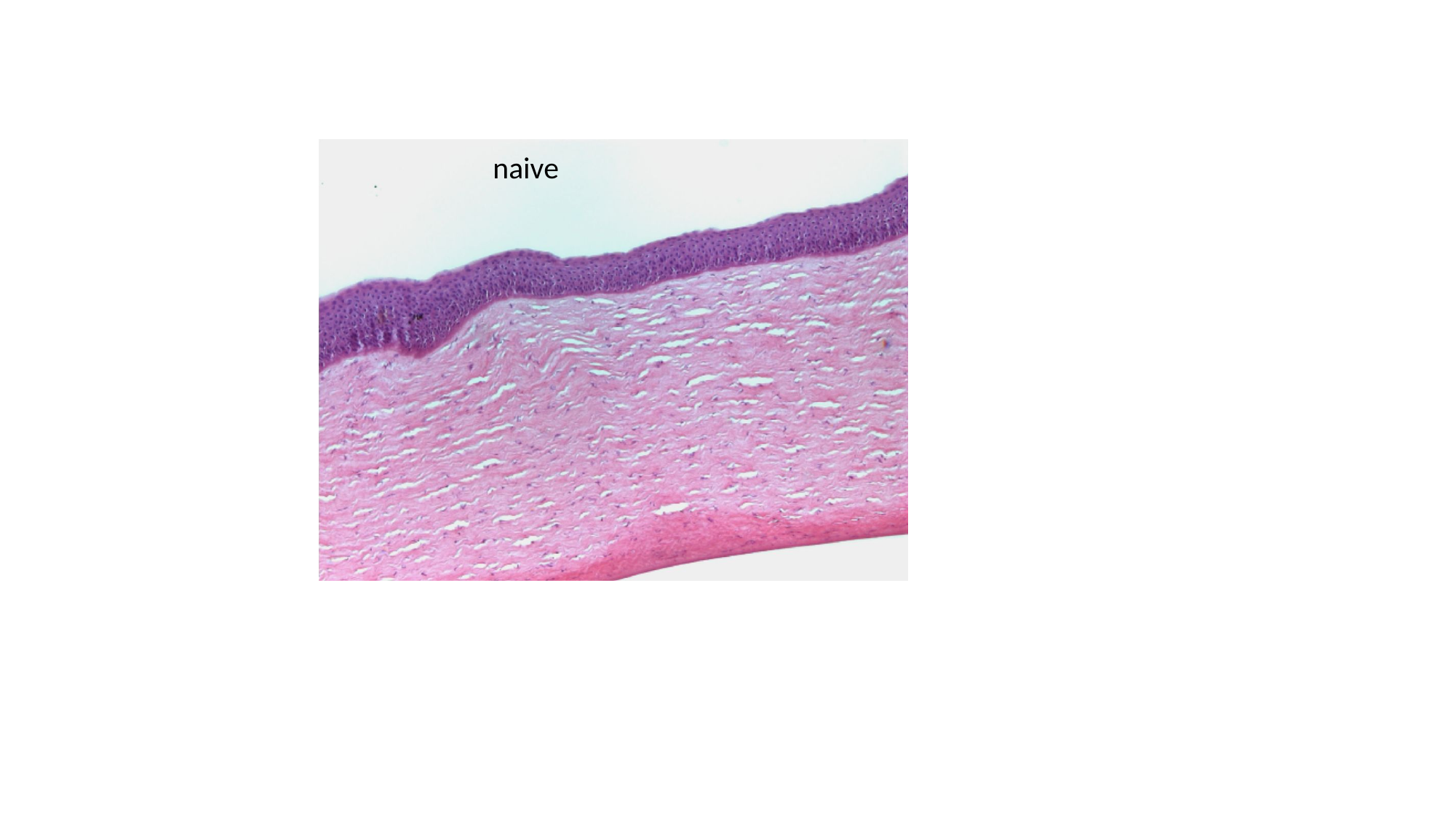

naive

Supplement: Source Data Figure 3 — Raw immunofluorescence tissue section images and raw hematoxylin and eosin stained tissue section images (raw, uncropped versions embedded in file). [file 41587_2022_1408_MOESM7_ESM.pptx]
